# Spatially resolved ex vivo drug response profiling in SMARCB1-deficient sinonasal carcinoma

**DOI:** 10.1038/s44321-026-00437-1

**Published:** 2026-05-02

**Authors:** Philipp Jurmeister, Susanne Flach, Linda Bergmayr, Konstanze Schleich, Edgar Chimal Calderon, Liliana H Mochmann, Yauheniya Zhdanovich, Doreen Klingler, Ada Pusztai, Anna Kübler, Christoph Walz, Christoph Benedikt Westphalen, Alexander Beck, Maximilian Leitheiser, Gerben E Breimer, Johannes A Rijken, Lot Devriese, Philipp Baumeister, Alena Skálová, Simon Schallenberg, Frederick Klauschen, Andreas Mock

**Affiliations:** 1 Institute of Pathology, Ludwig-Maximilians-University Munich, Munich, Germany; 2 German Cancer Consortium (DKTK), partner site Munich, a partnership between DKFZ and LMU Munich, Munich, Germany; 3 Department of Otorhinolaryngology, Head and Neck Surgery, Ludwig-Maximilians-Universität (LMU) Munich University Hospital, Munich, Germany; 4 Department of Medicine III and Comprehensive Cancer Center (CCC Munich LMU), LMU University Hospital, Munich, Germany; 5 Center for Neuropathology, Ludwig-Maximilian-University, Munich, Germany; 6 Institute of Pathology, Charité - Universitätsmedizin Berlin (Corporate Member of Freie Universität Berlin, Humboldt-Universität zu Berlin, and Berlin Institute of Health), Berlin, Germany; 7 Department of Pathology, University Medical Center Utrecht, Utrecht, The Netherlands; 8 Department of Head and Neck Surgical Oncology, University Medical Center Utrecht, Utrecht, The Netherlands; 9 Department of Medical Oncology, University Medical Center Utrecht, Utrecht, the Netherlands; 10 Bavarian Center for Cancer Research (BZKF), Munich Partner Site, Munich, Germany; 11 Department of Pathology, Charles University, Faculty of Medicine in Plzen, Plzen, Czech Republic; 12 Bioptic Laboratory, Ltd, Plzen, Czech Republic; 13 BIFOLD – Berlin Institute for the Foundations of Learning and Data, Berlin, Germany; 14 Division of Translational Medical Oncology, DKFZ & National Center for Tumor Diseases (NCT) Heidelberg, Heidelberg, Germany

**Keywords:** Cancer

## Abstract

SMARCB1-deficient sinonasal carcinoma (SDSC) is a rare, highly aggressive malignancy with limited therapeutic options and no established preclinical models. Here, single-nucleus RNA sequencing (snRNAseq), spatial transcriptomics, and ex vivo patient-derived tissue slice culture (TSC) were combined to resolve intratumoral heterogeneity, niche organization, and treatment vulnerabilities in an index SDSC. snRNAseq identified three malignant subpopulations, including two specialized states marked by ALDH1A1 and NTN4. Spatial profiling mapped these states to distinct niches. The ALDH1A1+ compartment localized to a basal-associated niche with intermingled p63-positive basal cells adjacent to stroma, showed reduced proliferative activity, and displayed stem-like transcriptional features. Ex vivo drug testing revealed a striking response: the mTOR inhibitor Sapanisertib induced extensive tumor necrosis and was associated with near-complete depletion of ALDH1A1+ and NTN4+ states, accompanied by strong stress/apoptosis signatures and reduced endothelial cells. In an additional retrospective cohort of 12 SDSC, ALDH1A1 was present in all cases with heterogeneous spatial patterns and higher levels in recurrences. Mesothelin was expressed in the index case and a subset of tumors, supporting mesothelin-directed therapeutic strategies.

## Synopsis

A multimodal pipeline combining snRNAseq, spatial transcriptomics and ex vivo tissue-slice culture maps cell states and drug responses in SMARCB1-deficient sinonasal carcinoma.

- Spatially resolved, ex vivo drug profiling was enabled in a rare sinonasal cancer using tissue-slice culture (TSC) paired to snRNAseq and spatial transcriptomics, linking treatment response to defined cell states.
- A stem-like ALDH1A1+ tumor cell population was identified and shown to occupy a basal-cell–associated niche, providing a mechanistic framework for how non-malignant epithelium may support tumor maintenance.
- Specific vulnerabilities were demonstrated by depletion of defined malignant subpopulations after mTORC inhibition (Sapanisertib) in the index tumor.
- High mesothelin expression was observed and validated by immunostaining, supporting therapeutic tractability with mesothelin-directed modalities in a subset of cases.
- A basal, stem-like epithelial compartment was identified as the most progenitor-like state and a potential cell of origin of SMARCB1-deficient sinonasal carcinoma.

## Abstract (web summary)

A multimodal pipeline combining snRNAseq, spatial transcriptomics and ex vivo tissue-slice culture maps cell states and drug responses in SMARCB1-deficient sinonasal carcinoma.

## Graphical abstract

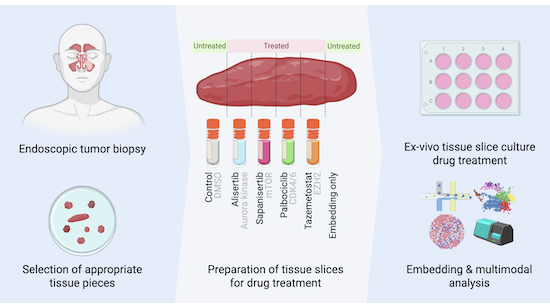

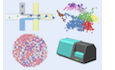

The paper explained **Problem**

SMARCB1-deficient sinonasal carcinoma (SDSC) is a rare, highly aggressive malignancy with poor clinical outcome and no established preclinical models. As a result, the mechanisms underlying intratumoral heterogeneity and potential therapeutic vulnerabilities in SDSC remain largely unknown.

**Results**

We combined single-nucleus RNA sequencing, spatial transcriptomics, sequential immunofluorescence, and ex vivo tissue slice culture to characterize SDSC at molecular, spatial, and functional levels. This revealed distinct malignant cell states, including an ALDH1A1-positive stem-like compartment and an NTN4-positive tumor population, each localized to specific spatial niches, enriched by non-malignant basal cells. Ex vivo drug testing identified marked sensitivity to Sapanisertib, which induced extensive necrosis and depleted both ALDH1A1-positive and NTN4-positive tumor cells. Validation in an independent cohort confirmed widespread ALDH1A1 expression, with higher levels in recurrent disease, and identified mesothelin expression in a subset of tumors.

**Impact**

Our study identifies a stem-like, spatially defined tumor compartment as a potential therapeutic vulnerability in SDSC and nominates mTOR inhibition as a promising treatment strategy. It also supports mesothelin as an additional candidate target. More broadly, this work establishes a multimodal ex vivo framework for biological discovery and therapy testing in rare cancers that lack conventional experimental models.

## Introduction

SMARCB1-deficient sinonasal carcinoma (SDSC) represents a rare and aggressive subtype of sinonasal malignancy, characterized by complete loss of INI1 protein expression due to biallelic deletion or truncating mutation of the corresponding *SMARCB1* gene (Bishop et al, 2014). Officially recognized as a distinct tumor entity in the 2017 WHO classification, SDSC constitutes ~3–5% of all sinonasal carcinomas (Llorente et al, 2014; Agaimy et al, 2017). SDSC presents significant diagnostic and therapeutic challenges due to its rarity, aggressive clinical course, high propensity for local recurrence, early metastatic spread, and overall poor prognosis, with 5-year survival rates typically ranging from only 30 to 50% (Jurmeister et al, 2022; Agaimy et al, 2017).

The molecular biology of SDSC revolves predominantly around the epigenetic (Chi et al, 2023) consequences of *SMARCB1* loss, notably dysregulation of the SWI/SNF chromatin remodeling complex and consequent upregulation of oncogenic pathways, including PRC2-driven gene silencing via EZH2 and dysregulation of cell-cycle regulators such as Cyclin D1 (Bishop et al, 2014; Agaimy et al, 2017). Despite clear molecular characterization of *SMARCB1* loss as the central pathogenic event, there remains a lack of established preclinical models for SDSC. To date, no robust cell lines, patient-derived xenografts, or organoid systems have been established, severely limiting functional characterization and translational research into potential therapeutic vulnerabilities (Danti et al, 2023).

Therapeutic approaches for SDSC have largely relied on extrapolation from protocols established for sinonasal undifferentiated carcinoma (SNUC), primarily involving aggressive surgery, radiotherapy, and platinum-based chemotherapy. However, outcomes remain unsatisfactory, and there is an urgent clinical need for novel targeted therapeutic strategies. Recent studies highlight potential vulnerabilities in SDSC that can be exploited pharmacologically, including inhibition of EZH2, CDK4/6, Aurora kinase, and mTOR pathways (Wilson et al, 2010; Cooper and Hong, 2022; Gounder et al, 2020). However, patient benefit has so far been very limited, potentially pointing to a complex tumor biology including intratumoral heterogeneity.

The introduction of single-cell and spatial transcriptomic technologies provides unprecedented opportunities to investigate tumor heterogeneity and molecular dynamics directly within the tumor microenvironment, especially relevant for rare entities lacking established models. However, such advanced methodologies have yet to be applied to SDSC. Here, we leveraged a unique clinical opportunity, obtaining fresh tissue from a confirmed case of SMARCB1-deficient sinonasal carcinoma, to perform a drug-response assay on ex vivo patient-derived tissue slice cultures (TSC) combined with comprehensive molecular analysis, including single-nucleus RNA sequencing and spatial transcriptomics.

## Results

### Molecular characterization and ex vivo drug testing

**Figure 1:**
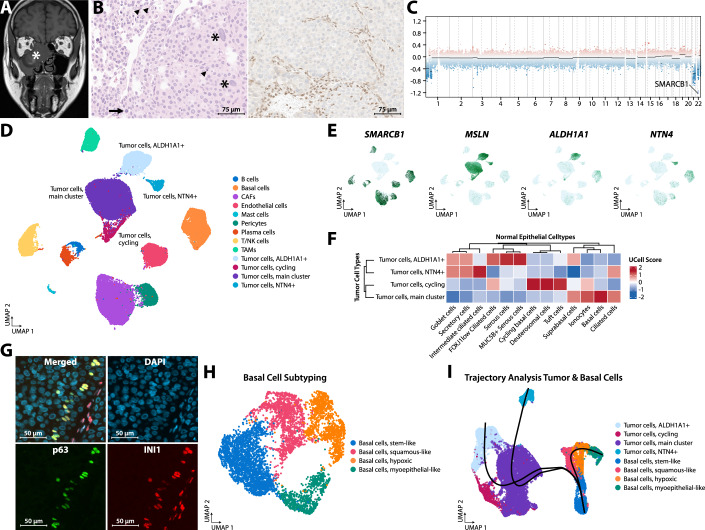
Integrated histological, molecular, and ex vivo characterization of a SMARCB1-deficient sinonasal carcinoma. (**A**) Magnetic resonance imaging of the index case demonstrating a tumor mass (asterisk) centered in the right nasal cavity with extension into the right paranasal sinuses and skull base. (**B**) Histomorphological and immunohistochemical characterization of the initial biopsy. Hematoxylin and eosin (H&E) staining reveals a tumor with a solid growth pattern, abundant eosinophilic cytoplasm, and round nuclei, some of which display marked pleomorphism (asterisks), mitotic figures (arrows), and apoptotic bodies (arrowheads). INI1 immunohistochemistry demonstrates a complete loss of nuclear expression in tumor cells, with preserved expression in surrounding stromal cells. (**C**) Genome-wide copy number profile derived from DNA methylation array data. A focal biallelic deletion is evident on chromosome 22, affecting the SMARCB1 locus. Apart from partial losses on chromosomes 1p and 21q, the overall copy number profile appears flat. (**D**) Uniform manifold approximation and projection (UMAP) visualization of the integrated single-nucleus RNA sequencing (snRNAseq) dataset. Four transcriptionally distinct tumor cell subclusters were identified: a main tumor cell cluster, a cycling subpopulation, an *ALDH1A1*-high cluster, and a cluster characterized by high *NTN4* expression. (**E**) Feature plots from the snRNAseq data highlighting expression of *SMARCB1*, *MSLN*, *ALDH1A1*, and *NTN4* across the tumor. (**F**) Heatmap summarizing UCell-based gene-signature scoring comparing tumor cell subclusters with a normal sinonasal epithelium reference. Main-cluster tumor cells showed the greatest similarity to basal epithelial signatures, whereas NTN4+ tumor cells most closely matched intermediate ciliated signatures and ALDH1A1+ tumor cells aligned with serous cell signatures, highlighting distinct differentiation-associated programs across tumor subclusters. (**G**) Sequential immunofluorescence imaging reveals basal cells interspersed among tumor cells. These basal cells retain nuclear INI1 expression, confirming their non-neoplastic nature, while INI1 expression is lost in tumor cells. TAMs tumor-associated macrophages, CAFs cancer-associated fibroblasts. (**H**) UMAP visualization of basal-cell subclustering revealed four distinct basal populations with stem-like, squamous-like, hypoxic, and myoepithelial-like features. (**I**) UMAP with Slingshot-based pseudotime/trajectory inference identified three lineages. One lineage connected stem-like basal cells to the main tumor cluster and progressed via cycling tumor cells toward the ALDH1A1+ tumor subcluster. A second tumor-associated lineage also passed through the main tumor cluster but diverged toward the NTN4+ tumor subcluster. A third lineage remained confined to the non-malignant basal epithelial compartment. Source data are available online for this figure.

We received a tumor specimen from a 57-year-old woman presenting with a lesion involving the nasal cavity and paranasal sinuses with involvement of the skull base (Fig. 1A), which was diagnosed at the Department of Otorhinolaryngology, Head and Neck Surgery at the LMU Klinikum Munich. Histomorphological features raised suspicion for a SWI/SNF complex-deficient sinonasal carcinoma, which was supported by immunohistochemical loss of nuclear INI1 (*SMARCB1*) expression (Fig. 1B). This diagnosis was further confirmed by a recently developed DNA methylation-based machine learning algorithm (Jurmeister et al, 2022), which classified the tumor as an SDSC. In line with this, copy number profiling derived from DNA methylation data revealed a loss of chromosome 22, including a focal biallelic deletion encompassing the *SMARCB1* gene locus, along with only minor additional alterations, specifically partial losses on chromosomes 1p and 21q (Fig. 1C).

A second biopsy obtained two weeks later provided additional material for both clinical molecular analyses and exploratory ex vivo drug testing. Bulk whole-exome sequencing revealed no clear driver mutation apart from a likely oncogenic deletion in the *CUBN* gene (*P833LfsX67*; Table EV1). Whole-transcriptome RNA sequencing detected no gene fusions but showed high expression of *MSLN* (Mesothelin; TPM = 137), which was discussed as a potential target for antibody-drug conjugate (ADC) therapy in the molecular tumor board. The tumor exhibited a low tumor mutational burden (0.71 mutations per megabase) and no evidence of homologous recombination deficiency (GIS score: 10).

The two largest tissue fragments of the second biopsy were selected for ex vivo patient-derived TSC preparation, and five slices were generated from each. The remaining tissue was formalin-fixed and paraffin-embedded (FFPE) for subsequent analyses. The first slice per fragment served as a DMSO-treated control, and four additional slices per tissue fragment were treated with the preselected inhibitors Sapanisertib (mTOR inhibitor), Alisertib (Aurora kinase inhibitor), Palbociclib (CDK4/6 inhibitor), and Tazemetostat (EZH2 inhibitor/epigenetic modulator). The remaining tissue was fixed and embedded for further analysis. Following ex vivo drug treatment for 48 h, all tissue slices were formalin-fixed and embedded for downstream histological and molecular analyses.

**Figure EV1:**
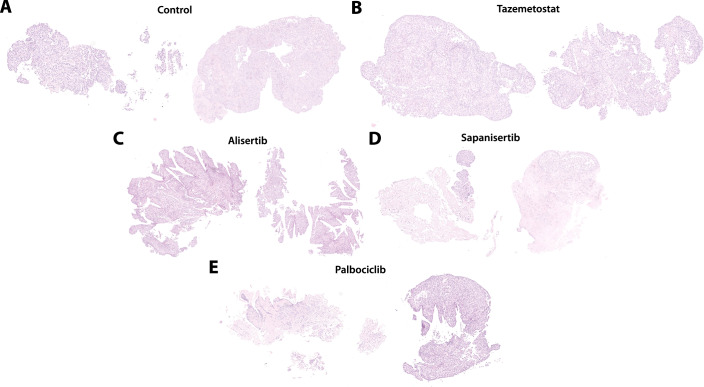
Hematoxylin and eosin (H&E) staining of patient-derived tissue slice cultures under different treatment conditions. (**A**) Control slices treated with the maximum equimolar concentration of DMSO. One of the control slices is also shown in Fig. 3B. (**B**) Tazemetostat-treated slices. (**C**) Alisertib-treated slices. (**D**) Sapanisertib-treated slices, showing extensive tumor necrosis in the lower portion of the tissue. One of the control slices is also shown in Fig. 3E. (**E**) Palbociclib-treated slices.

Histomorphological assessment confirmed the presence of representative tumor architecture in all embedded tissue specimens (Fig. EV1A–E). While loosely scattered apoptotic bodies were quite frequent in all samples, extensive areas of tumor necrosis were observed exclusively in the Sapanisertib-treated slices.

### Single-nucleus RNA sequencing

All embedded TSC specimens (including (a) uncultured control, (b) cultured but untreated control, and (c) treated samples) were subjected to single-nucleus RNA sequencing (snRNAseq). Data quality metrics were comparable across conditions, confirming that neither culture nor treatment affected assay performance. Cultured samples even showed marginally higher mean gene and UMI counts and a lower fraction of mitochondrial reads (Appendix Fig. S1).

Following joint preprocessing of all samples combined, marker gene-based cell type annotation identified all expected major tumor, immune and stromal cell populations (Fig. 1D; Appendix Fig. S2). Comparison between cultured and uncultured control tissue fragments revealed no substantial changes in overall cell type composition, supporting the biological representativeness and integrity of the TSCs (Appendix Fig. S3A).

Tumor cell clusters were identified based on loss of *SMARCB1* expression and—as known from bulk RNA sequencing—high expression of *MSLN* (Fig. 1E). High mesothelin expression was also confirmed on the protein level using seqIF (Appendix Fig. S4). Interestingly, we identified four transcriptionally highly distinct tumor cell subclusters. Importantly, these clusters were not associated with known technical confounders that can lead to spurious clustering (Appendix Fig. S3B–D). In addition to a main tumor cluster and a cycling tumor cell population, we observed two specialized subclusters, one marked by high *ALDH1A1* expression, and another characterized by strong *NTN4* expression (Table EV2). We further examined the expression of a broader panel of keratins, mucins, and cell-surface markers and identified clear differences between the tumor subclusters. NTN4+ and ALDH1A1+ tumor cells both showed high expression of *CEACAM6*, but otherwise displayed distinct epithelial programs: the NTN4+ subcluster was characterized by elevated *CD70*, whereas the ALDH1A1+ subcluster showed increased expression of *CD38*, *MUC4*, and *MUC15* (Appendix Fig. S5). To interpret these divergent differentiation-associated programs in the context of normal sinonasal epithelium, we applied gene-signature scoring and compared the tumor subclusters to a publicly available single-cell reference dataset of normal sinonasal epithelial cell types (Liao et al, 2025). In this analysis, main-cluster tumor cells showed the greatest similarity to basal cell signatures, whereas ALDH1A1+ tumor cells aligned most closely with serous cell signatures and NTN4+ tumor cells with intermediate ciliated signatures (Fig. 1F).

**Figure EV2:**
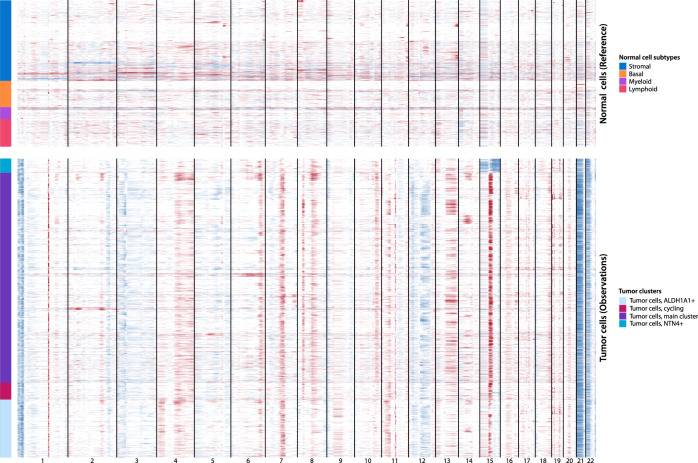
Heatmap of single-cell copy number profiles inferred from single-nucleus RNA sequencing data. Notably, loss of chromosome 15 is exclusively found in the NTN4+ tumor cell cluster.

We also detected a distinct basal-like epithelial cluster with high *TP63* (p63) expression. While variable p63 expression in immunohistochemistry has been reported in ~50% of SDSCs, prior studies described this expression specifically within tumor cells (Agaimy et al, 2017). In contrast, our snRNAseq data revealed that this basal cell cluster retained *SMARCB1* expression, lacked *MSLN* expression (Fig. 1E), and showed no alterations in copy number data inferred from snRNAseq data (Fig. EV2), clearly proving a non-neoplastic identity. Notably, this inference analysis also indicated loss of chromosome 15 as a unique feature of NTN4+ tumor cells. To also validate the non-neoplastic identity of basal cells on the protein level, we performed seqIF, which confirmed that basal cells indeed retained nuclear INI1 expression but were spatially intermingled with tumor cells (Fig. 1G), potentially forming a specialized tumor-associated niche.

Further subclustering of the basal cell cluster revealed four distinct cell states (Fig. 1H): (i) a stem-like basal cluster enriched for *LGR5* and *LGR6*, (ii) a squamous-like basal cluster enriched for *KRT6A* and *SPRR1B*, (iii) a hypoxic basal cluster characterized by *CA9*, *EGLN3*, *VEGFA*, and (iv) a myoepithelial-like basal cluster with elevated *SOX10*, *KRT14*, *MYLK*, and *ACTA2* expression (Appendix Fig. S6).

We then performed trajectory inference within the epithelial compartment (containing both basal and tumor cells) using Slingshot, selecting the stem-like basal cluster as the root because it showed the strongest stem/progenitor-associated expression profile. This analysis identified three trajectories in the epithelial compartment. One trajectory connected the stem-like basal cells to the main tumor cluster and then progressed through cycling tumor cells toward the ALDH1A1+ tumor cell cluster, while a second tumor-associated trajectory also passed through the main tumor cluster but diverged toward the NTN4+ tumor cell cluster. A third trajectory remained within the non-malignant basal epithelial compartment and extended from stem-like basal cells through squamous-like and hypoxic basal states toward the myoepithelial-like basal cluster.

### Spatial transcriptomic analysis

**Figure 2:**
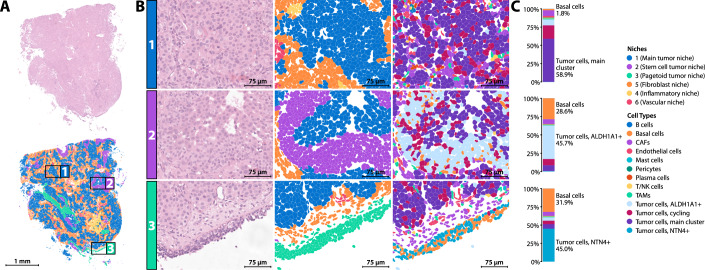
Spatial mapping of tumor cell subtypes and niche identification. (**A**) Overview of hematoxylin and eosin (H&E) staining and spatial transcriptomics data of an uncultured, untreated tissue section annotated with spatially inferred niches. Regions enriched for three distinct tumor-associated niches are highlighted (black boxes) and shown at higher magnification in (**B**). (**B**) H&E staining alongside spatial transcriptomics data displaying niche assignments and annotated cell types within representative tumor regions. Niche 1 was primarily composed of tumor cells from the main cluster (58.9%) and contained very few basal cells (1.8%). Niche 2 was enriched for ALDH1A1+ tumor cells (45.7%) and showed a high proportion of basal cells (28.6%). Niche 3 was predominantly composed of NTN4+ tumor cells (45.0%) and basal cells (31.3%), and was exclusively located in areas displaying pagetoid spread of tumor cells into pre-existing surface epithelium. (**C**) Stacked bar plot showing the relative proportions of all major cell types across the identified spatial niches. TAMs tumor-associated macrophages, CAFs cancer-associated fibroblasts.

To gain a more detailed understanding of the tumor’s spatial organization, we applied RCTD-based label transfer to map the previously identified cell types from snRNAseq onto a matched spatial transcriptomics dataset of uncultured, untreated tissue specimens (Fig. 2A,B). Spatial niche analysis revealed five distinct niches, each characterized by unique cell type compositions (Fig. 2C; Appendix Fig. S7; Table EV3):

- Niche 1 represented the primary tumor compartment, dominated by tumor cells from the main cluster (58.9%) and characterized by high proliferative activity (18.2%), while basal cells were nearly absent (1.8%).
- Niche 2 was enriched for ALDH1A1+ tumor cells (45.7%) and showed a notable presence of basal cells (28.6%). *ALDH1A1* is a well-established marker of cancer stem cells, and transcription factor activity analysis further supported this phenotype, revealing high activity of canonical stem cell regulators such as *KLF4* and *MEIS2* (Appendix Fig. S8). Additionally, we observed increased activity of transcription factors associated with retinoic acid signaling, including *RARA*, *RXRA*, and RXRB (Appendix Fig. S8). This aligns with high *ALDH1A1* expression, as *ALDH1A1* encodes an aldehyde dehydrogenase enzyme that catalyzes the conversion of retinaldehyde to retinoic acid, a critical metabolite in the regulation of cell fate, differentiation, and survival, including stem cell regulation. Furthermore, cycling tumor cells were significantly less frequent in Niche 2 compared to Niche 1 (8.6%; *p* < 0.001) and ALDH1A1+ tumor cells showed low activity of key proliferative transcription factors such as *E2F1* and *E2F3* (Appendix Fig. S8).
- Niche 3 was primarily composed of NTN4+ tumor cells (45.0%) and also displayed substantial enrichment of basal cells (31.9%). Similar to niche 2, the frequency of cycling tumor cells was significantly lower than in niche 1 (4.8%; *p* < 0.001). Histomorphological inspection revealed that niche 3 localized exclusively to regions where tumor cells exhibited pagetoid spread along the surface epithelium, a well-established histopathological feature of SDSC.
- Niches 4, 5 and 6 represented distinct tumor microenvironment niches. Niche 4 was predominantly composed of cancer-associated fibroblasts (CAFs), accounting for 53.1% of the respective niche populations. Niche 5 exhibited marked enrichment of immune cells, including B cells (15.3%), T/NK cells (21%), plasma cells (6.4%), and tumor-associated macrophages (TAMs; 11.5%), representing a more immune-infiltrated environment. Niche 6, by contrast, was characterized by a high abundance of endothelial cells (38%) and pericytes (16.2%), consistent with a vascular niche.

### Results from ex vivo tissue slice culture drug testing

As outlined above, histopathological assessment of H&E-stained slides revealed apoptotic bodies across all samples, but extensive tumor cell necrosis was observed exclusively in Sapanisertib-treated tissue slices.

**Figure 3:**
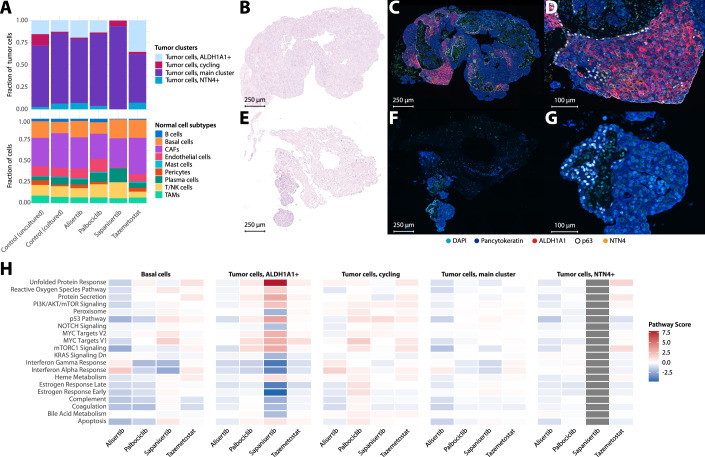
Sapanisertib treatment selectively eliminates stem-like tumor cells and substantially reduces endothelial cells. (**A**) Stacked bar plots show the distribution of tumor cell subclusters (top) and normal cell subtypes (bottom) across all conditions, based on single-nucleus RNA sequencing data. A near-complete loss of ALDH1A1 and NTN4-positive tumor cells is observed in the Sapanisertib-treated sample. Endothelial cells also markedly decrease (0.6% vs. a median of 6.9% in other samples) while comparable pericyte cell counts can be observed. (**B**) Representative hematoxylin and eosin (H&E) section of a cultured, untreated control tissue slice, showing preserved tumor morphology without extensive necrosis. This image is also shown in Fig. EV1A. (**C**) Low-magnification sequential immunofluorescence (seqIF) image of the same sample, with tumor cells identified by pancytokeratin (dark blue). ALDH1A1-positive tumor cells are prominently seen in red. (**D**) Higher magnification of panel (**C**) highlights ALDH1A1-positive tumor cells intermingled with p63-positive basal cells (white) at the basal layer. Scattered superficial NTN4+ tumor cells (orange) are also visible. (**E**) H&E of a tissue slice treated with Sapanisertib, showing widespread tumor cell necrosis. This image is also shown in Fig. EV1D. (**F**) Overview seqIF image of a Sapanisertib-treated sample. No ALDH1A1 or NTN4 signal is detected. (**G**) Higher magnification of panel F. While interspersed p63-positive basal cells are still detectable, confirming tissue representativity, ALDH1A1 and NTN4 positive tumor cells are entirely absent. TAMs tumor-associated macrophages, CAFs cancer-associated fibroblasts. (**H**) Heatmap showing pathway activity changes for the top ten upregulated and top ten downregulated Hallmark pathways identified in ALDH1A1+ tumor cells treated with Sapanisertib. Values for Sapanisertib treatment effects in NTN4+ tumor cells could not be calculated since they were no longer present after treatment.

Detailed analysis of the cellular composition further demonstrated a marked reduction or complete absence of ALDH1A1+ (0.3 vs. 12.8% in the control) and NTN4+ tumor cells (0 vs. 6.3% in the control) following Sapanisertib treatment (Fig. 3A). In contrast, basal cells were present at comparable levels (15.2 vs. 12.8% in the control), excluding sampling bias as an explanation for the loss of ALDH1A1+ and NTN4+ tumor cells. The only other notable shift in tumor cell composition was observed in Tazemetostat-treated samples, which showed an increase in ALDH1A1+ tumor cells (35.5 vs. 12.8% in the control), accompanied by a reduction in tumor cells from the main cluster (54.5 vs. 79.2%). With regards to non-malignant cells, no obvious shifts between cultured or uncultured control and the treated tissue slices was observed, with the exception of a strikingly reduced number of endothelial cells in tissue slices treated with Sapanisertib (0.6% vs. median 6.9% in the other samples) while Pericytes were not affected.

**Figure EV3:**
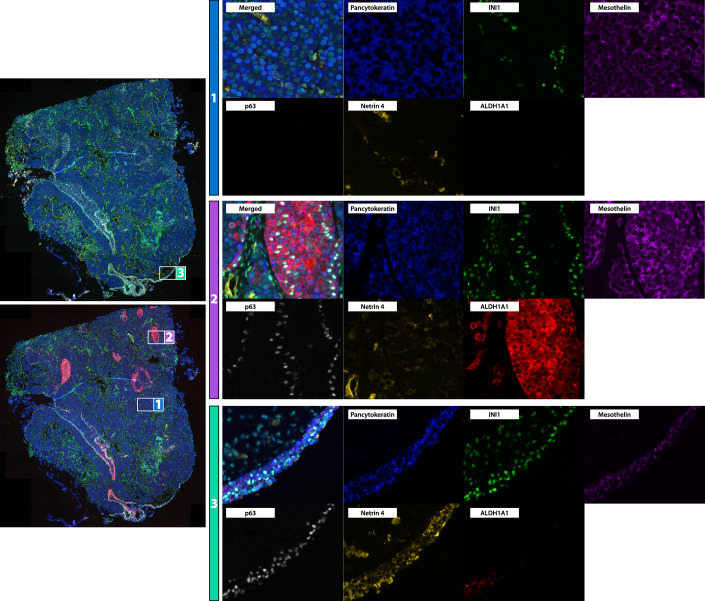
Validation of tumor niches identified by spatial transcriptomics on the protein level. Overview and high-magnification sequential immunofluorescence images of a cultured, untreated tissue slice culture recapitulate the three spatial tumor niches defined by spatial transcriptomic analysis. Niche 1 represents the main tumor compartment, characterized by an absence of p63-positive basal cells. NTN4 expression is confined predominantly to endothelial cells lining blood vessels, with only rare expression in tumor cells, and ALDH1A1 is absent. Niche 2 is composed of ALDH1A1-high tumor cells intermingled with p63-positive basal epithelial cells. Netrin 4 again localizes mainly to endothelial cells, retaining nuclear INI1 expression, while tumor cell staining is infrequent. Niche 3 illustrates pagetoid spread of INI1-negative, pancytokeratin-positive tumor cells within the surface epithelium, showing strong Netrin 4 expression and complete absence of ALDH1A1.

To validate the effect of Sapanisertib treatment at the protein level, we established a seqIF marker panel (pancytokeratin, INI1, ALDH1A1, NTN4, p63, and mesothelin) to enable standardized assessment of the previously identified tumor niches. seqIF staining on both an uncultured, untreated tissue specimen (Fig. EV3) as well as a cultured, untreated control (Fig. 3B–G) was able to confirm the identified niches on the protein level. In contrast, while Sapanisertib-treated samples retained both tumor and basal cells, ALDH1A1- and NTN4-positive tumor cells were completely absent (Fig. 3E–G).

**Figure EV4:**
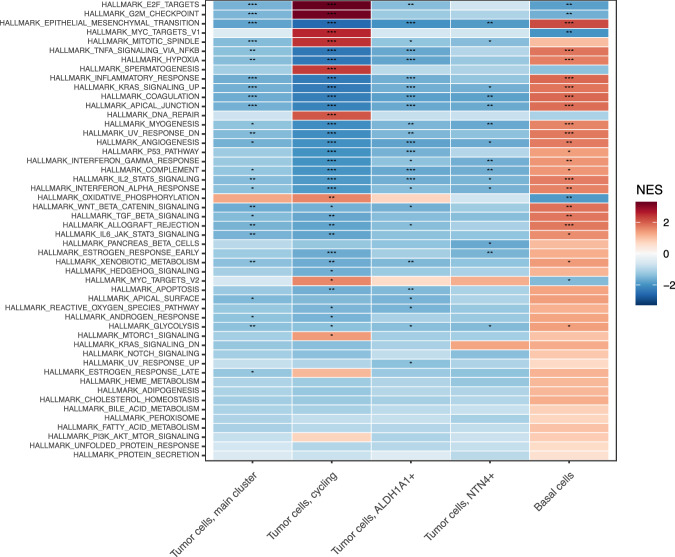
Heatmap of baseline Hallmark pathway activity score differences between basal and tumor cells in untreated, uncultured control samples.

To gain mechanistic insight into the selective loss of ALDH1A1+ tumor cells following Sapanisertib treatment, we performed single-cell pathway activity analysis. In untreated control samples, baseline pathway activity patterns were broadly similar across tumor subclusters and basal cells, with expected enrichment of cell-cycle-associated programs in cycling tumor cells (Fig. EV4). In particular, we did not observe consistent baseline enrichment of mTOR/PI3K pathway activity in ALDH1A1+ or NTN4+ tumor cells.

In contrast, under Sapanisertib treatment, ALDH1A1+ tumor cells showed the most pronounced and selective changes in pathway activity compared with both the cultured control and the other tumor subclusters (Fig. 3H and Appendix Fig. S9). Upregulated programs included apoptosis/p53-associated responses and oxidative stress signatures, together with strong induction of unfolded protein response/protein handling pathways consistent with ER/proteostasis stress. At the same time, pathway activity signatures annotated to mTOR/PI3K signaling were increased, consistent with a potential feedback response to mTOR pathway inhibition. Conversely, estrogen-response-associated programs and NOTCH signaling activity were reduced in Sapanisertib-treated ALDH1A1+ cells.

### Validation of the tumor stem cell niche in an independent cohort of SMARCB1-deficient sinonasal carcinomas

To determine whether the concept of an *ALDH1A1*+, potentially therapeutically vulnerable tumor stem cell niche is a generalizable feature of SDSC or a tumor-specific phenomenon of this specific patient, we analyzed an independent cohort of 12 primary SMARCB1-deficient sinonasal tumor samples, including both SMARCB1-deficient carcinomas and adenocarcinomas, a recently proposed variant (Skálová et al, 2024). In addition, material from recurrent disease was available for two of these cases (cases 11, 11R and 12, 12R), including a local recurrence in the nasal cavity as well as a cervical lymph node metastasis.

**Figure 4:**
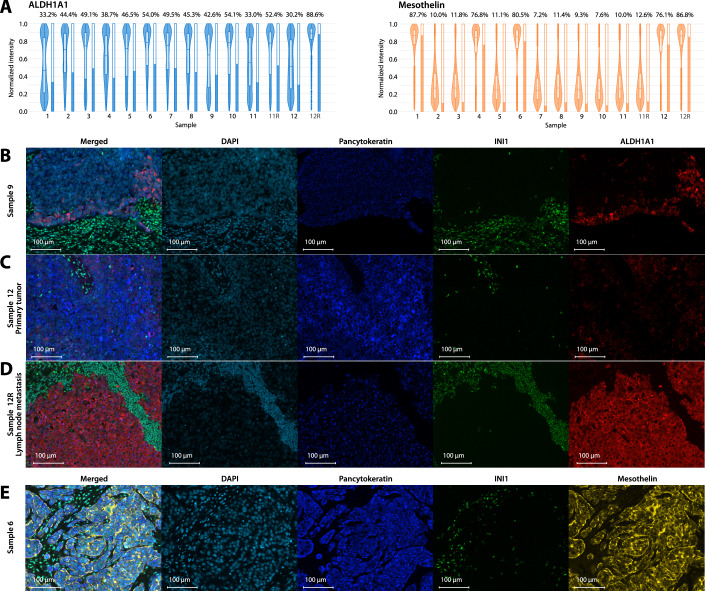
Sequential immunofluorescence (seqIF) analysis of the retrospective cohort. (**A**) Combined violin and bar plots showing normalized intensity values and the proportion of positive cells for ALDH1A1 and mesothelin across all samples. The numbers of cells included were as follows: Sample 1, *n* = 40,213; Sample 2, *n* = 27,190; Sample 3, *n* = 33,413; Sample 4, *n* = 20,564; Sample 5, *n* = 52,420; Sample 6, *n* = 48,851; Sample 7, *n* = 34,255; Sample 8, *n* = 46,512; Sample 9, *n* = 50,208; Sample 10, *n* = 24,526; Sample_11, *n* = 27,046; Sample 11 R, *n* = 38,129; Sample 12, *n* = 38,892; and Sample 12 R, *n *= 26,192. Box plots are shown with the center line indicating the median, the box limits representing the 25th and 75th percentiles (interquartile range), and the whiskers extending to the minimum and maximum values. (**B**–**D**) Representative merged and single-channel seqIF images illustrating different spatial distribution patterns of ALDH1A1 expression. Tumor cells are identified by pancytokeratin positivity and INI1 loss. In Sample 9 (**B**), ALDH1A1 expression was predominantly restricted to a basal tumor cell layer adjacent to the stroma. A similar region of the same sample is also shown in Fig. EV5B. Sample 12 (**C**) showed scattered, weakly ALDH1A1-positive cells without clear spatial organization. The recurrent sample from the same patient (Sample 12 R, **D**; lymph node metastasis) displayed diffuse and strong ALDH1A1 expression in nearly all tumor cells. (**E**) Merged and single-channel images from Sample 6 showing strong mesothelin expression in almost all tumor cells, with negative staining in stromal cells (pancytokeratin-negative, INI1-positive nuclei). Source data are available online for this figure.

ALDH1A1-expressing tumor cells were present in all samples, with proportions ranging from 33.0 to 88.6% (Fig. 4A). Three patterns of spatial distribution were observed: (1) predominant localization in a “basal” tumor cell layer adjacent to surrounding stroma (Fig. 4B); (2) groups of positive cells intermixed between negative cells without clear spatial organization (Fig. 4C); (3) diffuse expression in almost all tumor cells (Fig. 4D). Patterns (1) and (2) were not mutually exclusive and could be found in different regions of the same tumor. For the matched primary and recurrent samples, we consistently observed significantly higher ALDH1A1 expression (*p* < 0.001) in the recurrent sample (Fig. 4A,C,D).

**Figure EV5:**
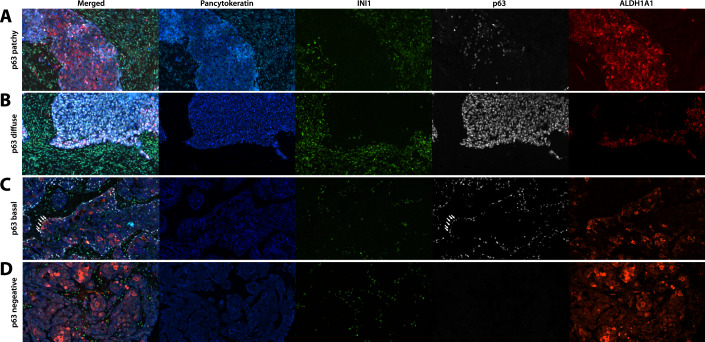
Representative staining patterns of p63 in the retrospective cohort. (**A**) Patchy expression of p63 in tumor cells (loss of nuclear INI1). (**B**) Diffuse p63 expression in almost all tumor cells. A similar region of the same sample is also shown in Fig. 4B. (**C**) Predominant expression of p63 in tumor cells at the basal (near stroma; white arrows) layer. (**D**) No p63 expression in tumor cells.

In contrast to the index case, none of the additional SDSC cases showed intermingling of p63-positive non-neoplastic basal cells within the tumor. Instead, p63 immunoreactivity in tumor cells was observed in 7/14 cases (50%), a frequency in line with previous reports.(Bishop et al, 2014) We observed three distinct patterns: strong, diffuse nuclear staining in essentially all tumor cells (three cases; Fig. EV5A), patchy staining in a subset of tumor cells (two cases; Fig. EV5B), or predominantly basal/peripheral staining within tumor nests (two cases; Fig. EV5C). The remaining 7/14 cases (50%) were p63-negative (Fig. EV5D). Importantly, ALDH1A1 expression was not associated with p63 status or pattern and was observed in both p63-positive and p63-negative tumors.

Weak to moderate NTN4 expression was detected in a subset of tumor cells (4.3–10.5%) across all samples, but without clear spatial organization. SeqIF images of a representative case are shown in Appendix Fig. S10. None of the retrospective cohort samples exhibited pagetoid spread into the surface epithelium, preventing assessment of whether NTN4 expression would be more prominent in this specific tumor cell population.

Furthermore, strong mesothelin expression could be confirmed in four out of twelve patients (33%; Fig. 4A,E).

## Discussion

SDSC remains a rare, highly aggressive cancer with poor prognosis, limited therapeutic evidence, and no established preclinical models, underscoring the urgent need for biologically informed treatment strategies. Here, we combine multimodal profiling - deep snRNAseq, spatial transcriptomics and seqIF - with an organotypic ex vivo tumor TSC platform to dissect spatial tumor-microenvironment heterogeneity and test drug responses in native tissue. To our knowledge, this constitutes the most comprehensive molecular and functional characterization of SDSC to date and the first demonstration of ex vivo drug testing on an organotypic model of this disease.

Our snRNAseq data revealed heterogeneous malignant compartments, including a main tumor cluster and a cycling subpopulation, as well as two specialized subsects: an ALDH1A1*+* population and an NTN4*+* population. Comparison with a normal sinonasal epithelial reference further supported a broad differentiation spectrum, with each tumor cell cluster showing greatest transcriptional similarity to distinct normal epithelial programs. Together, these findings are compatible with the hypothesis that SDSC arises in a progenitor-like epithelial context with basal characteristics and retains sufficient plasticity to adopt differentiation-associated features of multiple sinonasal epithelial lineages. In our dataset, the stem-like basal cell cluster most closely matched such a progenitor-like state, and trajectory inference supported a branching organization in which ALDH1A1+ and NTN4+ tumor cells occupy divergent endpoints, consistent with distinct differentiation-associated programs. This interpretation is further supported by inferred copy number profiles indicating a chromosome 15 loss uniquely enriched in the NTN4+ subcluster, suggestive of subclonal evolution. While pseudotime/trajectory analyses provide computational ordering of transcriptional states rather than definitive lineage directionality, these results motivate a model in which stem-like basal cells represent a plausible cell of origin for SDSC with subsequent diversification.

Spatial mapping localized ALDH1A1*+* tumor cells to a basal layer adjacent to stroma, forming a discrete niche enriched for basal (TP63+) cells. This aligns with ALDH1 family activity as a hallmark of cancer stem-like states across multiple tumor types (Ginestier et al, 2007) and with the concept that stem-like tumor cells often adopt a quiescent or slow-cycling phenotype that is under-represented in proliferative fractions (Santos-de-Frutos and Djouder, 2021). In line with this, ALDH1A1+ cells showed reduced proliferative transcription factor activity (e.g., *E2F1*/*E2F3*), while transcriptional programs implicated in stemness and retinoid biology (e.g., *KLF4*, *MEIS2*, and *RARA/RXRA*/*RXRB*) were enriched. The colocalization with *TP63*-expressing basal cells suggests that a tumor-associated, regeneration-mimicking niche may support stem-biased states in SDSC (Li et al, 2023), potentially shaping disease course and treatment resistance.

Functionally, the TSC drug screen indicated a potential selective vulnerability to the mTOR kinase inhibitor Sapanisertib: treated slices displayed extensive tumor necrosis and a near-complete loss of both ALDH1A1+ and NTN4+ tumor cells, while basal cells were retained at comparable levels - arguing against sampling bias. In parallel, endothelial cells were markedly reduced, consistent with the anti-angiogenic effect of mTOR pathway inhibition within the tissue (Guba et al, 2002). While baseline pathway activity profiling in untreated controls did not reveal prominent enrichment of mTOR/PI3K-related pathways in ALDH1A1+ or NTN4+ tumor cells relative to other malignant subclusters, striking differences emerged upon treatment. ALDH1A1+ tumor cells showed the most pronounced transcriptional response to Sapanisertib, consistent with proteotoxic stress, a well-known effect of mTOR inhibition (Chou et al, 2012; Appenzeller-Herzog and Hall, 2012). Concurrent suppression of pro-survival signaling and reduced NOTCH pathway activity further supports disruption of stemness-associated programs in this subpopulation. Notably, mTOR/PI3K gene sets were among the most elevated pathways after treatment, a pattern that may reflect compensatory feedback within the PI3K/AKT/mTOR axis. For example, S6K1-dependent inhibitory phosphorylation of IRS-1 has been reported to be induced upon mTOR inhibition, which can result in reactivation of upstream PI3K/AKT signaling (O’Reilly et al, 2006).

Together, these data suggest that the ALDH1A1+ compartment occupies a therapeutically vulnerable niche and is closely spatially associated with basal cell and vascular compartments. The pronounced response to Sapanisertib and the observed transcriptional changes following treatment are consistent with involvement of the mTOR/PI3K axis, although we cannot exclude additional indirect or off-target contributions to the observed effects.

Importantly, we observed strong ALDH1A1 protein expression across an independent SDSC cohort, with three spatial patterns (basal-layer-enriched, intermixed foci, and diffuse). In two patients with matched longitudinal samples, ALDH1A1 expression was significantly higher in recurrences than in primary tumor samples, supporting a model in which this compartment endures initial therapy and may seed regrowth. Although our ex vivo testing did not include chemotherapeutic drugs, prior work links high ALDH1A1 expression to a broad tolerance via detoxification and retinoid metabolism (Ginestier et al, 2007; Santos-de-Frutos and Djouder, 2021). Our data suggests that targeting mTOR signaling can debulk this compartment in the index case TSC model within its native niche. In early clinical trials, combination strategies of Sapanisertib with taxane-based chemotherapy, metformin or other targeted therapies are already being explored in different tumor types (Tao et al, 2025; Han et al, 2023; Subbiah et al, 2023). Collectively, these findings support mTOR pathway inhibition as a candidate vulnerability that warrants validation in additional cases.

Beyond state-directed therapy, our integrated multi-omic analyses uncovered additional biology-informed opportunities. Bulk RNA sequencing identified high mesothelin expression in the index case, and seqIF confirmed a strong expression in a subset of the validation cohort. Mesothelin has several established oncogenic functions: it enhances proliferation and survival by activating PI3K and AKT signaling (Bharadwaj et al, 2011), promotes invasion via MMP-9 upregulation (Wang et al, 2012), and polarizes tumor-associated macrophages toward an immunosuppressive M2-like phenotype (Dangaj et al, 2011). Interestingly, mesothelin expression in normal tissues is restricted to mesothelial linings (Hassan et al, 2020). Given the maturing clinical landscape for mesothelin-directed antibody-drug conjugates (ADCs) and other targeted compounds, mesothelin may offer an orthogonal therapeutic development in SDSC (Hassan et al, 2016; Wang et al, 2025).

Beyond its disease-specific insights, this study exemplifies how ex vivo TSCs can serve as a powerful complement to other model systems, such as 3D cell culture organoids. Unlike conventional cultures derived from dissociated cells, TSCs preserve the native architecture, stromal and immune compartments, and vascular organization of tumors (Gerlach et al, 2014; Donnadieu et al, 2016; Greier et al, 2023). This intact spatial environment enables simultaneous multimodal readouts directly within the patient’s own tissue context. As demonstrated in this work, such integrative analyses allow the identification of clinically relevant subclones, stromal interactions, and niche-specific drug responses that may be lost in reconstituted models.

We acknowledge limitations. The discovery dataset is centered on a single index tumor reflecting the extreme rarity of SDSC. While our independent cohort supports generalizability of *ALDH1A1* expression patterns and mesothelin positivity, functional validation across additional cases and with expanded drug panels—including chemotherapies and rational combinations—will be essential. Furthermore, while spatial analyses and endothelial depletion suggest niche-level mechanisms, causality (e.g., whether vascular changes mediate part of the anti-tumor effect) warrants dedicated perturbation studies. In particular, it remains unclear whether the spatial organization with non-malignant basal cells intermingled with ALDH1A1+ tumor cells observed in the index case is essential for the proposed sensitivity to Sapanisertib. As this specific pattern was not consistently observed in other tumors from the retrospective cohort, their response to treatment may, in principle, differ.

Furthermore, the relatively short incubation period may have been insufficient to capture the full effects of some tested compounds—particularly Tazemetostat, whose activity as an epigenetic modulator might require longer exposure. Likewise, potential adaptive or resistance mechanisms may not have had time to emerge within this timeframe.

In sum, our data support a model in which an ALDH1A1+, stem cell-like malignant compartment localizes to a basal, stroma-proximal niche, withstands initial cytotoxic stress, and contributes to recurrence. Therapeutically, two potentially complementary strategies emerge: (i) state-directed approaches to weaken or eradicate the stem cell-like clone (e.g., mTOR pathway inhibition), and (ii) target-directed approaches leveraging recurrent surface markers such as mesothelin with modern ADCs. Given the lethality of SDSCs and the scarcity of models, these findings—while early—provide a concrete biological framework and actionable signals to prioritize in further preclinical studies.

## Methods

**Table:** Reagents and tools table

Reagent/resource	Reference or source	Identifier or catalog number
**Experimental models**		
Human: SMARCB1-deficient sinonasal carcinoma (SDSC) biopsy-derived tissue slice culture (TSC)	This study (index case)	N/A
Human: SDSC FFPE validation cohort (*n* = 12)	This study (retrospective cohort)	N/A
**Antibodies**		
Anti-INI1 (SMARCB1) antibody	Ventana, USA	MRQ-27; RRID not available
Pancytokeratin antibody	Agilent	Clone AE1/AE3; 1:100; RRID AB_2132885
Anti-SMARCB1 antibody	Cell Signaling Technology	Clone D8M1X; 1:100; RRID AB_2800172
Anti-Mesothelin antibody	Cell Signaling Technology	Clone D9R5G; 1:600; RRID AB_2800323
Anti-p63 antibody	Cell Signaling Technology	Clone D9L7L; 1:50; RRID AB_2799159
Anti-ALDH1A1 antibody	Cell Signaling Technology	Clone D9Q8E; 1:200; RRID AB_2799452
**Chemicals, enzymes and other reagents**		
Sapanisertib (mTORC1/2 inhibitor)	Selleck, USA	L1300
Palbociclib (CDK4/6 inhibitor)	Selleck, USA	L1300
Alisertib (Aurora kinase A inhibitor)	Selleck, USA	L1300
Tazemetostat (EZH2 inhibitor)	Selleck, USA	L1300
Lunaphore Multistaining buffer kit	Lunaphore, Switzerland	BU06
Lunaphore Elution buffer kit	Lunaphore, Switzerland	BU07-L
Lunaphore Quencing buffer kit	Lunaphore, Switzerland	BU08-L
Lunaphore Imaging buffer kit	Lunaphore, Switzerland	BU09-L
**Software**		
R	R Project	N/A
Seurat/SeuratObject	Hao et al, 2024; Seurat	N/A
Harmony	Korsunsky et al, 2018	N/A
spacexr	Cable et al, 2022	N/A
decoupleR	Badia-i-Mompel et al, 2022	N/A
PROGENy	Schubert et al, 2018	N/A
CollecTRI	Müller-Dott et al, 2023	N/A
UCell	Andreatta and Carmona, 2026	N/A
Slingshot	Street et al, 2018	N/A
QuPath	Bankhead et al, 2017	N/A
conumee	Daenekas et al, 2024	N/A
**Other**		
Countess 3 Automated Cell Counter	Thermo Fisher Scientific, USA	N/A
Tissue-Tek automated staining system	Sakura Finetek, Japan	N/A
BenchMark XT staining platform	Ventana, USA	N/A
Lunaphore Comet Chips	Lunaphore, Switzerland	MK03

### Patients and samples

Fresh tumor tissue from the index case was retrieved using transnasal biopsy under local anesthesia. Additional leftover material from routine diagnostics of the same tumor was retrieved from the archive of the Institute of Pathology, LMU Munich. Written informed consent was obtained from the index patient prior to sample collection, which was approved by the local ethics committee of the LMU Klinikum in Munich, Germany (ref. nos. 18-446 and 25-0629) as well as the data protection officer. The study participant consented to sharing pseudonymized data and samples within and outside of the European Union. The study has been conducted in accordance with the Declaration of Helsinki and in keeping with the rules of good clinical practice and according to the German laws and ethical standards.

For validation of key findings, a retrospective cohort of additional Formalin-fixed, paraffin-embedded (FFPE) samples from SDSC was assembled from the archives of the Departments of Pathology at Charité—Universitätsmedizin Berlin, University Medical Center Utrecht and Bioptical Laboratory Pilzen.

### Magnetic resonance imaging (MRI)

Representative clinical MRI images (Fig. 1A) were acquired on a Siemens MAGNETOM Aero 1.5 T scanner (Siemens Healthineers, Germany) using a coronal T1-weighted spin-echo sequence. Acquisition parameters were: TR 526 ms, TE 8.8 ms, matrix 320 × 320, slice thickness 3 mm, slice spacing 3.3 mm.

### Histochemistry and immunohistochemistry

Hematoxylin and eosin (H&E) staining was performed on an automated Tissue-Tek staining system (Sakura Finetek, Japan) according to the manufacturer’s instructions. Immunohistochemical analysis for INI1 was performed on an automated BenchMark XT staining platform (Ventana, USA) using the ready-to-use MRQ-27 antibody (Ventana, USA, RRID not available).

### DNA methylation analysis

DNA methylation profiling was performed using the Illumina Infinium MethylationEPIC v2 BeadArray (Illumina, USA) following standard protocols. Arrays were scanned on an Illumina NextSeq 550 system, and copy number profiles were derived using a modified version of the Conumee package (v2.0) (Daenekas et al, 2024). DNA methylation-based classification was performed using a web-based classification platform (https://epiverse.io).

### Bulk whole-exome- and transcriptome-sequencing

FFPE tumor tissue was processed as part of the national genomic medicine framework (Modellvorhaben Genomsequenzierung, Germany) using a standardized whole-exome and transcriptome sequencing workflow. Tumor areas with representative pathology were isolated by microdissection. DNA and RNA were extracted using the QIAamp DNA FFPE Advanced and RNeasy Kits (Qiagen, Germany), respectively, and germline DNA was obtained from EDTA blood with the QIAamp DNA Micro Kit (Qiagen, Germany). Nucleic acid concentrations were measured with the Qubit system, and RNA was reverse transcribed to cDNA. Tumor cell content was ~80%.

Three complementary next-generation sequencing (NGS) assays were performed: paired whole-exome sequencing (WES), targeted germline analysis, and RNA exome sequencing. Libraries were prepared from tumor and matched germline DNA using the Twist Exome v2 Panel Kit (TWIST Bioscience, USA; 36.5 Mb target) and sequenced on an Illumina NovaSeq X Plus system. Data processing, including demultiplexing, alignment (hg19), variant calling, and assessment of TMB, HRD, and MSI, was performed with the Illumina DRAGEN Bio-IT Platform v4.3 (“Somatic WES Tumor Normal” and “Germline WES” modules). Variants were annotated using VEP (Ensembl), ClinVar, and the BRCA Exchange database. Classification followed ACMG and VICC guidelines via the Franklin-Genoox database.

Clinically relevant variants (gnomAD ≤0.01, exonic ±30 bp, ClinVar classes 4–6, or pathogenic BRCA Exchange entries) and loss-of-function mutations in tumor suppressor genes (TSGene database) were prioritized. Workflow performance was validated within the German Network for Personalized Medicine (Menzel et al, 2024).

RNA exome sequencing (TWIST Bioscience) was conducted to profile transcriptome-wide expression and fusion events. Analysis using the Illumina DRAGEN v4.2 RNA pipeline (BCL-to-FASTQ conversion, alignment, BAM/BAI generation) and ARRIBA (Uhrig et al, 2021) enabled detection of fusion transcripts, which were annotated using MANE, HAVANA, UCSC, and COSMIC databases. Only clinically relevant, in-frame fusions or loss of regulatory elements were reported.

### Tissue slice generation

After retrieval, tissue samples were immediately transferred into MACS Tissue Storage Solution (Miltenyi Biotec, Germany) and transported at 4 °C. Within 4 h, tissue slices of two separate tissue pieces were prepared using a vibrating microtome (Compresstome VF-310-0Z, Precisionary, USA). Following careful removal of excess surface liquid, each tissue piece was mounted on a sample holder and circumferentially embedded in 2% agarose (LE Agarose, Biozym, Germany) within the processing tube. Slices of 300 µm thickness were generated (settings: oscillation 5.5, speed 5) and subsequently cultivated at the air–liquid interface in 12-well plates with membrane inserts (0.4 µm PET clear extended culture plate, cellQUART, Germany). The tissue left on the sample holder after cutting was fixed in formalin and paraffin-embedded to serve as a culture-naive control tissue. On day 1 of cultivation, cultures were maintained in DMEM/F12 basal medium supplemented with 1% penicillin/streptomycin and 1% amphotericin B. After 24 h the amphotericin B concentration was reduced to 0.1% alongside the addition of drugs.

### Tissue slice culture treatment

Two tissue slices each were treated with Sapanisertib, Palbociclib, Alisertib, or Tazemetostat (Selleck, USA) at concentrations of 0.1, 0.5, 1, and 5 µM. As controls, two additional slices were incubated with 500 µM DMSO alone, matching the highest concentration present in the drug dilutions. Both treated and control samples were incubated for 48 h, then fixed in formalin and paraffin-embedded (FFPE) for subsequent analyses.

### Sample preparation of fixed tissue slice culture specimens

After fixation and embedding, samples were sectioned to generate an H&E-stained slide. All stained sections were reviewed by a board-certified pathologist, confirming the presence of representative tumor tissue in each sample. Owing to limited tissue availability, one FFPE block was used to generate one additional section reserved for potential sequential immunofluorescence (seqIF). The remaining material from this block, together with all tissue from the second block processed under the same conditions, was pooled to obtain sufficient nuclei/cell numbers for single-nucleus RNA sequencing (snRNAseq) analysis.

Uncultured, untreated control specimens yielded more material and—in addition to H&E sections—were used to generate additional sections for Xenium spatial transcriptomics as well as multiple blank slides reserved for seqIF.

None of the investigators were blinded to the identity of the samples analyzed in the study.

### Single-nucleus RNA sequencing

Samples were processed in fourfold multiplex experiments. To obtain nuclei, all available FFPE tissue was cut into at least ten 25-μm slides. Deparaffinization and tissue dissociation were performed according to the 10X Genomics manual on the gentleMACS Octo Dissociator (Miltenyi Biotec, Germany).

The number of intact nuclei was approximated with a Readycount Green/Red Cell Viability Stain (Thermo Fisher, USA) on the Countess 3 Cell Counter (Thermo Fisher, USA; Table EV4). In addition, nuclei preparations were assessed by visual inspection of the counter images prior to downstream processing to confirm intact nuclear morphology and to exclude preparations with excessive debris or nuclear aggregation/clumping. The hybridization protocol was modified based on improved performance observed in previous experiments: samples were incubated at least 16 h at 42 °C with an additional 2 h hold step at 23 °C after the first 30 min of incubation. All subsequent steps were performed as outlined in the manual option A—pooled wash workflow. A filtering step was only performed at the end of the workflow; the filter was washed with Post-Hyb buffer to obtain an optimal yield of available nuclei. Afterward, an additional centrifugation step (850 rcf, 5 min) was performed to remove the excess buffer before counting again. For pipetting the GEM chip, an excess of 20% compared with the recommendation of the pooled cells was used when enough viable nuclei were available. The sample index PCR was performed with ten cycles for 4-plexed samples and eight cycles for 16-plexed samples to achieve a maximum targeted cell recovery. Library concentrations were determined with a 1xds DNA HS Assay Kit on a Qubit4 Fluorometer (Thermo Fisher, USA) and then measured with the DNA High Sensitivity reagents on an Agilent 1200 Bioanalyzer (Agilent Technologies, USA) to determine the average fragment length. Libraries were sequenced on an Illumina NovaSeq X Plus System using a 1.5B flow cell with 200 reads. Demultiplexing was performed using the on-board BCLconvert application.

### Spatial transcriptomics analysis

Paraffin-embedded tissue slices were cut into 4.5-µm-thick slices, put in cold water for hydration and fit on Xenium slides in a 38 °C water bath. Slides were processed according to the Xenium Prime In Situ Gene Expression User Guide (CG000760, Revision A) on a VFW XT96 (VWR Avantor, USA) thermocycler. The Xenium Prime 5 K Human Pan Tissue & Pathway Panel was used, including the optional cell segmentation kit. Buffers were prepared according to the Xenium Analyzer User Guide. Sample plate A was centrifuged for 10 min at 4 °C at 1600 rcf. (CG000584, Revision J, 10X Genomics). Slides were imaged on the Xenium Analyzer (10X Genomics, USA; v4.0.1.4).

### Sequential immunofluorescence

Sequential immunofluorescence was performed on a Lunaphore Comet 1.0 device (Lunaphore, Switzerland). 2 µm tissue sections were air-dried and deparaffinized in a hybridization oven at 60 °C for 1 h. Antigen retrieval was carried out using Lab Vision Dewax and HIER buffer H (Epredia, USA) by immersing slides in preheated buffer (85 °C) within a PT-module (Epredia, USA) and subsequently heating to 102 °C for 1 h. Staining was performed in five consecutive cycles:

- Cycle 1: NTN4 (polyclonal, 1:200, Biotechne, RRID not available)
- Cycle 2: Pancytokeratin (clone AE1/AE3, 1:100, Agilent, RRID AB_2132885) and SMARCB1 (clone D8M1X, 1:100, Cell Signaling Technology, RRID AB_2800172)
- Cycle 3: Mesothelin (clone D9R5G, 1:600, Cell Signaling Technology, RRID AB_2800323)
- Cycle 4: p63 (clone D9L7L, 1:50, Cell Signaling Technology, RRID AB_2799159)
- Cycle 5: ALDH1A1 (clone D9Q8E, 1:200, Cell Signaling Technology, RRID AB_2799452)

Secondary antibodies included Alexa Fluor Plus 555 donkey anti-goat (polyclonal, 1:200, Thermo Fisher, USA) for NTN4; Alexa Fluor Plus 555 goat anti-mouse (polyclonal, 1:200, Thermo Fisher, USA) for pancytokeratin; and Alexa Fluor Plus 647 goat anti-rabbit (polyclonal, 1:400, Thermo Fisher Scientific) for SMARCB1, mesothelin, p63, and ALDH1A1.

All buffers were prepared according to the manufacturer’s User Manual (version 001, Lunaphore, Switzerland). The standard FFPE tissue run template was adapted as follows: (i) no blocking step was performed, (ii) a quenching step was included before each cycle (30 s), (iii) primary antibody incubation was 4 min for NTN4 and ALDH1A1 and 8 min for Pancytokeratin, INI1, Mesothelin, and p63, (iv) secondary antibody incubation was 2 min per cycle, and (v) each cycle ended with a 2-min elution step. All procedures were carried out at 37 °C.

### Processing of snRNAseq and spatial transcriptomics data

FASTQs from snRNAseq analysis were processed using CellRanger (10X Genomics, USA; v9.0.1) with standard settings. Filtered feature-barcode matrices were imported into Seurat (v5.2.1) (Hao et al, 2024). Cells with fewer than 400 or more than 6000 detected genes, more than 15,000 UMI counts, or >10% mitochondrial gene content were excluded. Data were log-normalized with Seurat, variable features were identified (2000 per dataset), and data were scaled before principal component analysis. To minimize batch effects between cultured and uncultured conditions, Harmony (v1.2.3) (Korsunsky et al, 2018) was applied with group.by.vars = “culture_status” to regress out effects caused by tissue culturing alone. Uniform Manifold Approximation and Projection (UMAP) embeddings were generated on the first 30 Harmony-corrected principal components, followed by nearest-neighbor graph construction and clustering using the Louvain algorithm at a resolution of 0.3. Major cell compartment clusters were annotated using canonical marker genes (Appendix Fig. S1) and differentially expressed features identified using the FindMarkers function implemented in Seurat. To refine annotations, stromal, tumor, basal, macrophage, and T-cell subsets were reclustered individually using the same preprocessing pipeline. Cells with mixed expression profiles indicative of multiplets or high ambient RNA contamination were excluded from further analysis.

Pathway activity was inferred using PROGENy implemented via decoupleR (v2.12.0), while transcription factor activity was estimated using the CollecTRI network (Schubert et al, 2018; Badia-i-Mompel et al, 2022; Müller-Dott et al, 2023). Per-cell scores were stored as Seurat assays and summarized at the cluster or sample level. Differences between conditions were tested using Wilcoxon tests with FDR correction.

For comparison of tumor and basal cell populations with normal sinonasal epithelial cell types, we used a publicly available preprocessed Seurat object from Liao et al (Liao et al, 2025). The external reference and our dataset were merged and restricted to the intersecting gene set shared between the two platforms (3′ versus probe-based). Batch effects were corrected using Harmony. Similarity between normal epithelial cell types in the external reference and the tumor/basal populations in our dataset was assessed by gene-signature scoring using UCell (Andreatta and Carmona, 2026).

Pseudotime and trajectory analysis was performed using Slingshot (v2.14.0) with “Basal cells, stem-like” as a root cluster (Street et al, 2018).

Matched Xenium spatial transcriptomics data were processed in Seurat. Cell type labels derived from snRNAseq and transferred using robust cell type decomposition (RCTD) implemented in the spacexr package (v2.2.1) (Cable et al, 2022), with Seurat-derived references mapped onto Xenium cell centroids. Spatial niches were defined using BuildNicheAssay (parameters: niches.k = 6, neighbors.k = 30). Cell-type composition of niches was quantified and compared as absolute and relative frequencies.

### Processing of sequential immunofluorescence data

Background subtraction on raw image data were performed using Horizon Viewer (Lunaphore, Switzerland; v2.3.0.0). Exported OME TIFF files were loaded in QuPath (v.0.60) (Bankhead et al, 2017). Cell segmentation was performed using the QuPath Cell Detection feature with standard parameters. Detection measurements were saved as a tab delimited file and imported into R. The data table was filtered and structured for further processing using R base functions. For INI1 and p63, signal intensity values for the nucleus compartment and for the rest of the samples from the cytoplasm compartment were selected for further analysis. To normalize marker intensities and control for differences in staining and dynamic range, each marker’s intensity values were normalized using percentile-based normalization. To binarize marker expression, each marker’s normalized intensities were assessed using Gaussian mixture modeling (GMM) to detect a distinct positive subpopulation. The two-component GMM was selected if it improved model fit (based on BIC) compared to a single-component model. If GMM fitting failed or was indistinguishable, a fallback threshold at the 95th percentile was applied. Cells in the higher-intensity cluster were labeled positive.

### Graphics

The visual abstract was created using BioRender.com.

## Supplementary information

Table EV1

Table EV2

Table EV3

Table EV4

Appendix

Peer Review File

Source data Fig. 1

Source data Fig. 4

Expanded View Figures

## Data Availability

Raw data generated in this study has been deposited in Zenodo under DOI accession 10.5281/zenodo.19201425 (https://zenodo.org/records/19201425). The source data of this paper are collected in the following database record: biostudies:S-SCDT-10_1038-S44321-026-00437-1.
