# Supplementary material for: Spatially resolved ex vivo drug response profiling in SMARCB1-deficient sinonasal carcinoma
Source: EMBO Mol Med. 2026 May 2;18(6):2360–78. doi: 10.1038/s44321-026-00437-1 (PMC13270136; doi:10.1038/s44321-026-00437-1)
Supplement: Supplementary file 5 — Appendix [file 44321_2026_437_MOESM5_ESM.pdf]

Appendix

Table of Contents

Appendix Figure S1 2

Appendix Figure S2 2

Appendix Figure S3 3

Appendix Figure S4 3

Appendix Figure S5 3

Appendix Figure S6 4

Appendix Figure S7 4

Appendix Figure S8 5

Appendix Figure S9 6

Appendix Figure S10 6

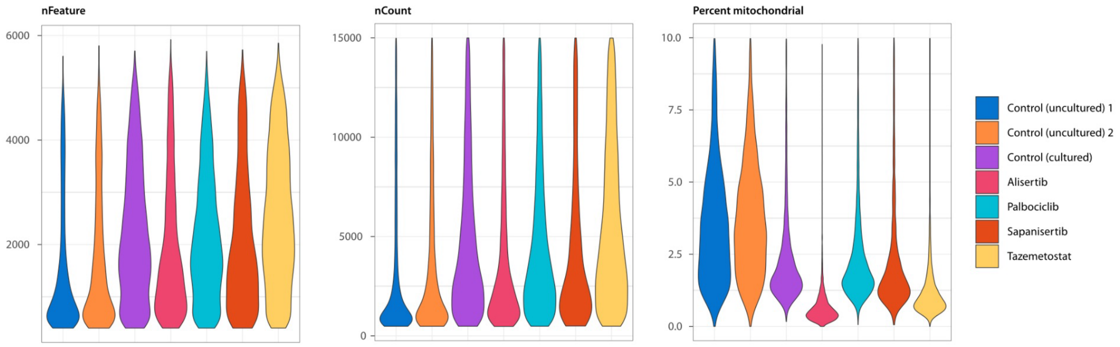

Appendix Figure S1
Quality control metrics of cultured and uncultured tissue slice samples.
Violin plots showing the distribution of detected genes per nucleus (nFeature; **A**), total UMI counts (nCount; **B**), and mitochondrial read fraction (**C**) across uncultured controls, cultured control (DMSO treated), and drug-treated tissue slices.

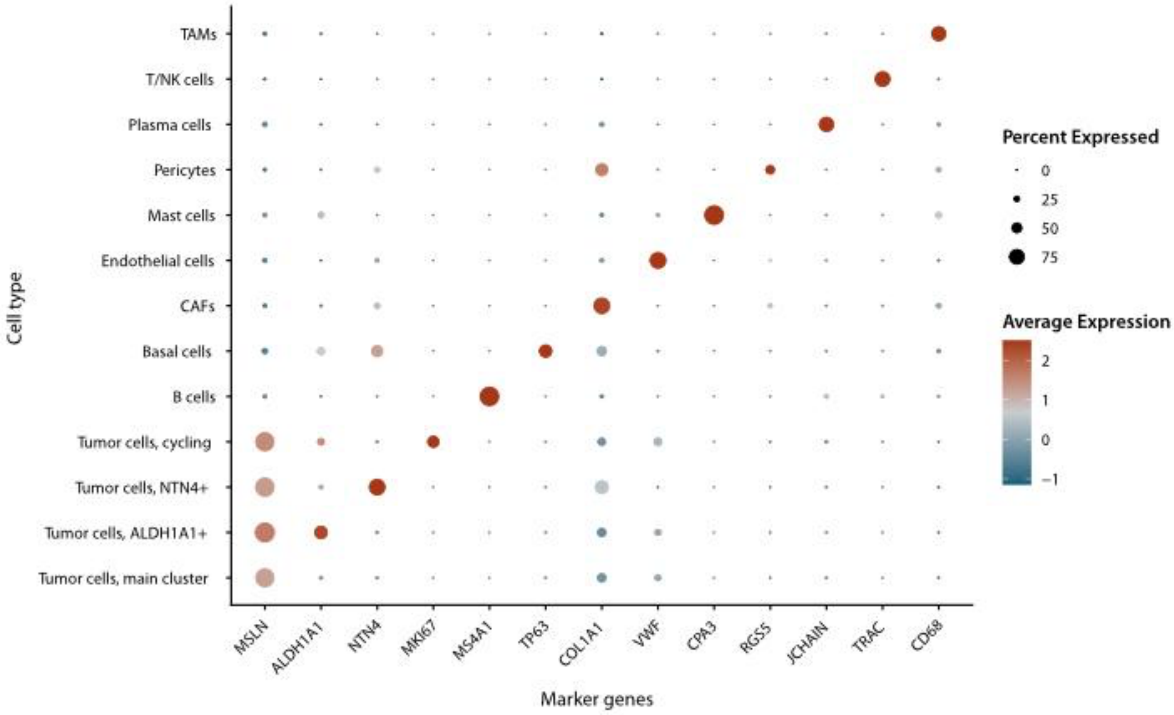

Appendix Figure S2
Dot plot showing marker gene expression of major cell types identified by single nucleus RNA sequencing.
Abbreviations: TAMs = tumor-associated macrophages; CAFs: cancer-associated fibroblasts

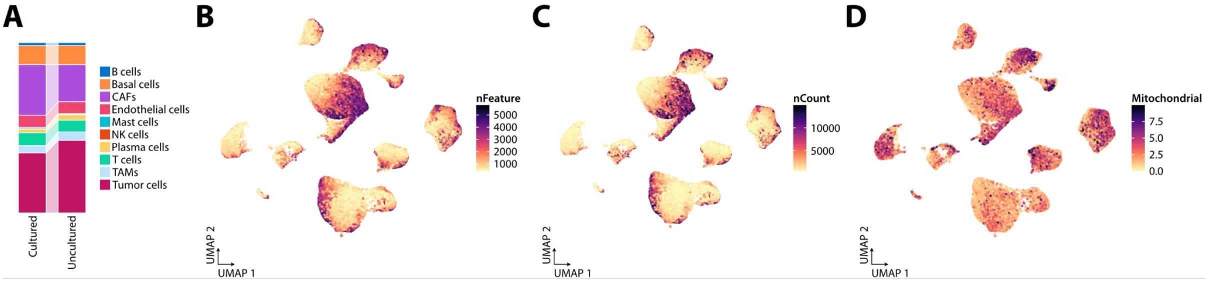

Appendix Figure S3

Comparison of tissue composition of treated and untreated samples as well as Uniform Manifold Approximation and Projection (UMAP) visualization of potential confounders.

A Stacked bar plot depicting global cell type composition in cultured versus uncultured tissue fragments. Comparable distributions indicate that short-term ex vivo culture maintains the native cellular architecture of the tumor microenvironment.

B UMAP plot colored by the number of detected features per cell.

C UMAP plot colored by the total number of counts per cell.

D UMAP plot colored by the percentage of mitochondrial reads per cell.

Abbreviations: TAMs = tumor-associated macrophages; CAFs: cancer-associated fibroblasts

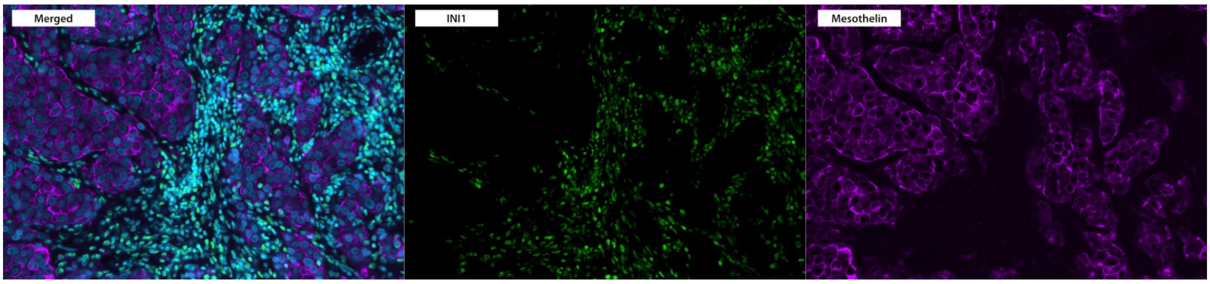

Appendix Figure S4

Representative sequential immunofluorescence images of an uncultured, untreated control specimen from the index case. Consistent with the single-nucleus RNA sequencing findings, tumor cells (identified by loss of nuclear INI1) showed strong mesothelin protein expression, whereas stromal fibroblasts and immune cells with retained nuclear INI1 showed no detectable mesothelin staining.

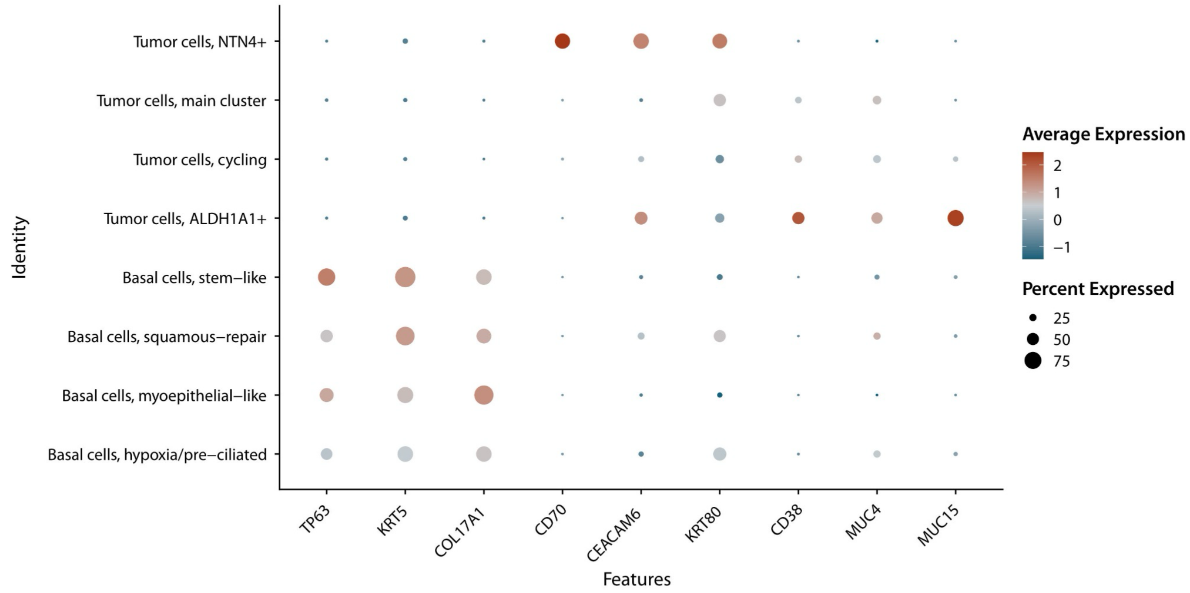

Appendix Figure S5

Dotplot showing the expression of selected keratins, mucins and cell surface markers between different tumor and basal cell clusters.

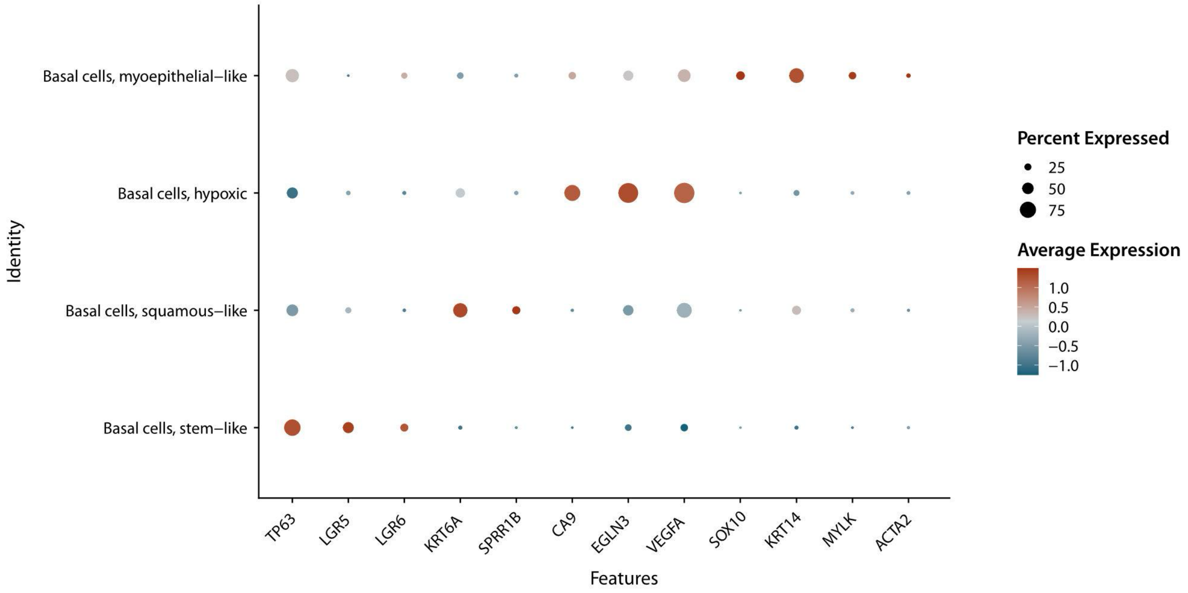

Appendix Figure S6
Dotplot showing the expression of characteristic marker genes of the different basal cell subclusters.

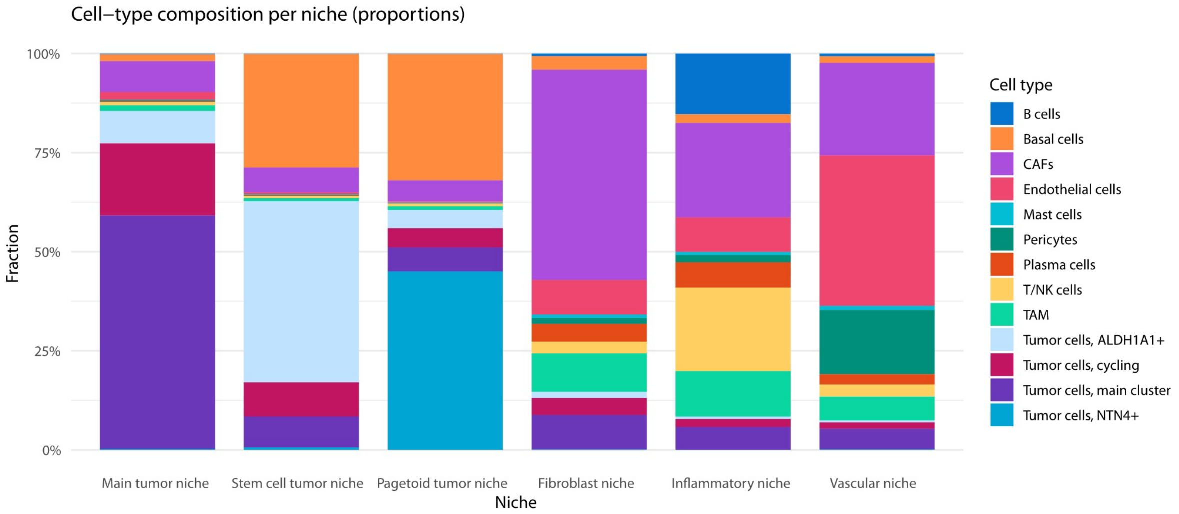

Appendix Figure S7
Stacked bar plot illustrating the cell type composition of each spatial niche. Niche 1 corresponds to the main tumor niche, predominantly composed of tumor cells from the major clusters. Niche 2 represents a stem cell-like niche characterized by enrichment of ALDH1A1-positive tumor cells and basal cells. Niche 3 reflects the pagetoid spread in the surface epithelium, with enrichment of NTN4-positive tumor cells and basal cells. Niche 4 is enriched in stromal components, particularly cancer-associated fibroblasts (CAFs) while Niche 5 also contains multiple immune cell types, including B cells and T cells, representing an inflammatory niche. Niche 6 constitutes a vascular niche, enriched by endothelial cells and pericytes.

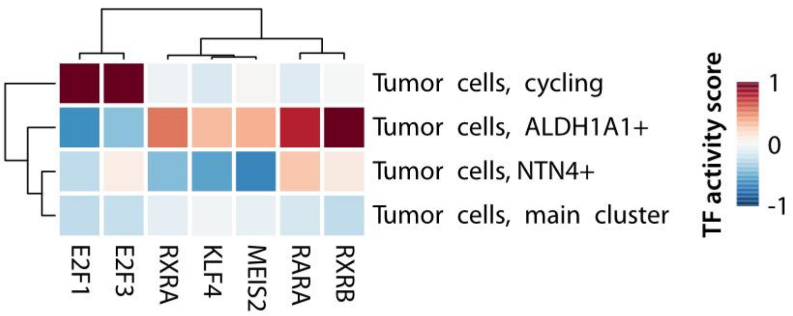

Appendix Figure S8

Results from transcription factor (TF) activity inference. ALDH1A1+ tumor cells exhibited strong activation of stem cell-associated TFs *KLF4* and *MEIS2*, as well as enhanced activity of retinoid metabolism-related regulators *RXRB*, *RARA*, and *RXRA*, while showing reduced activity of proliferation-associated factors *E2F1* and *E2F3*.

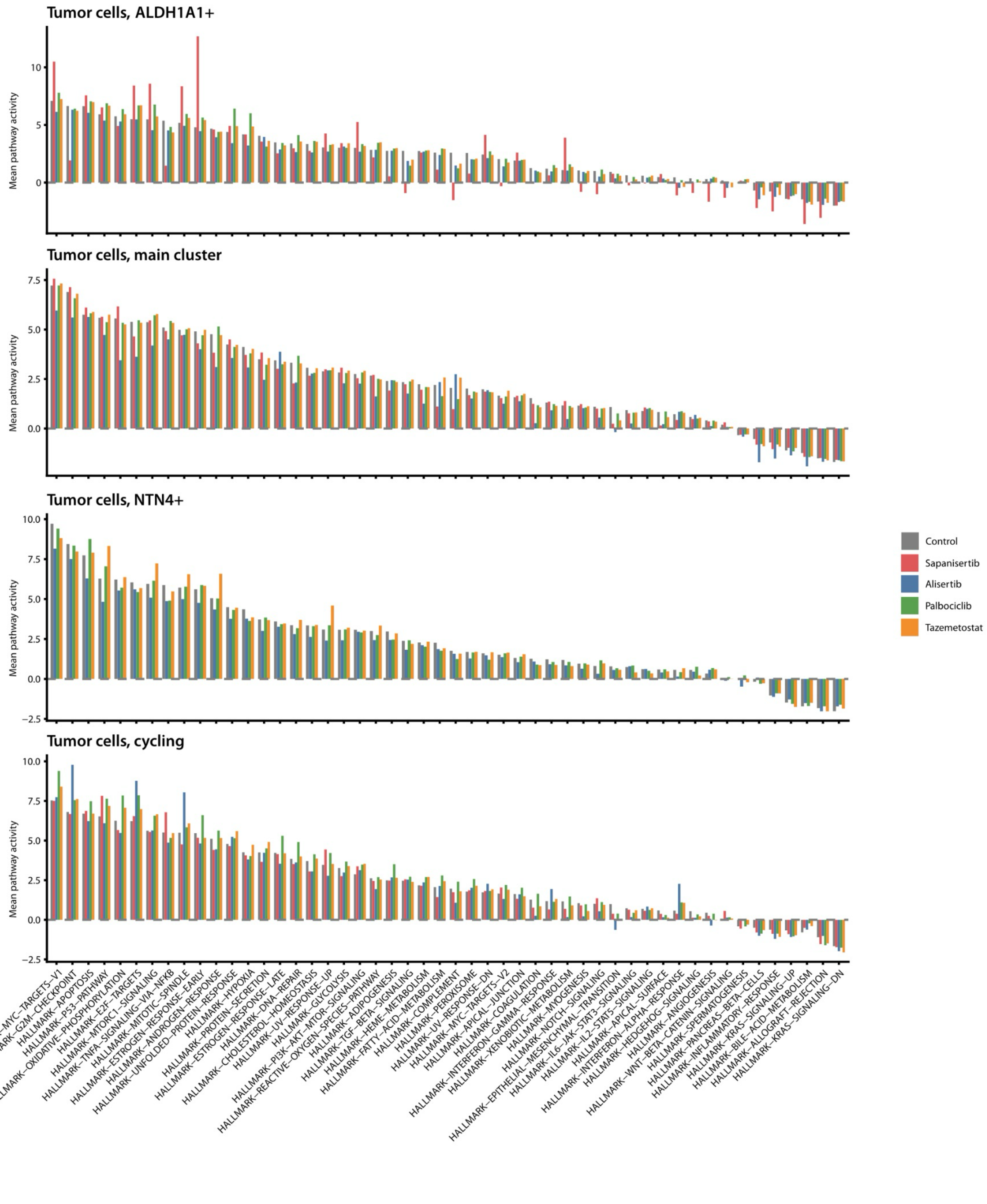

Appendix Figure S9

Barplots visualizing pathway activity scores in different tumor cell clusters under different treatment conditions. While most tumor cell clusters are little affected by the used inhibitors, ALDH1A1+ tumor cells show drastic changes following Sapanisertib treatment.

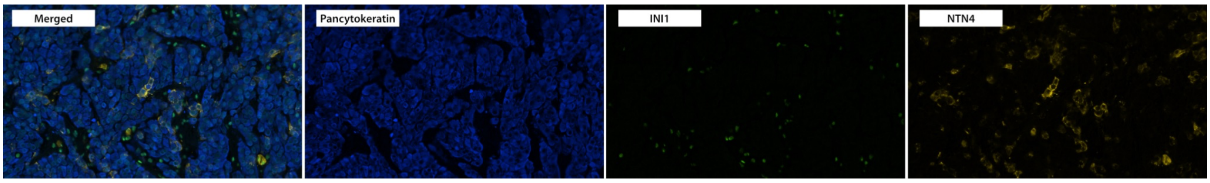

Appendix Figure S10

Representative, patchy NTN4+ expression in tumor cells in the retrospective cohort without clear spatial organization.
