# Supplementary material for: Spatially resolved ex vivo drug response profiling in SMARCB1-deficient sinonasal carcinoma
Source: EMBO Mol Med. 2026 May 2;18(6):2360–78. doi: 10.1038/s44321-026-00437-1 (PMC13270136; doi:10.1038/s44321-026-00437-1)
Supplement: Supplementary file 9 — Expanded View Figures [file 44321_2026_437_MOESM9_ESM.pdf]

Expanded View Figures

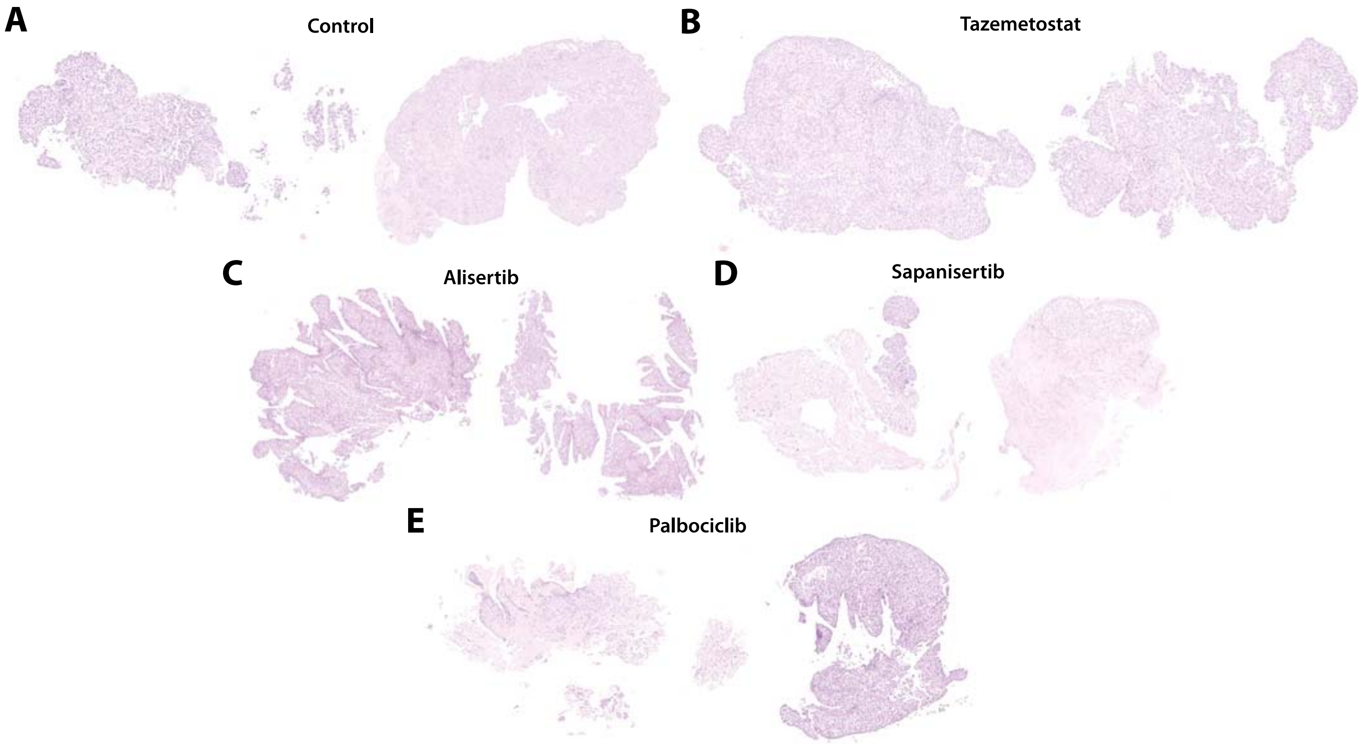

Figure EV1. Hematoxylin and eosin (H&E) staining of patient-derived tissue slice cultures under different treatment conditions.

(**A**) Control slices treated with the maximum equimolar concentration of DMSO. One of the control slices is also shown in Fig. 3B. (**B**) Tazemetostat-treated slices. (**C**) Alisertib-treated slices. (**D**) Sapanisertib-treated slices, showing extensive tumor necrosis in the lower portion of the tissue. One of the control slices is also shown in Fig. 3E. (**E**) Palbociclib-treated slices.

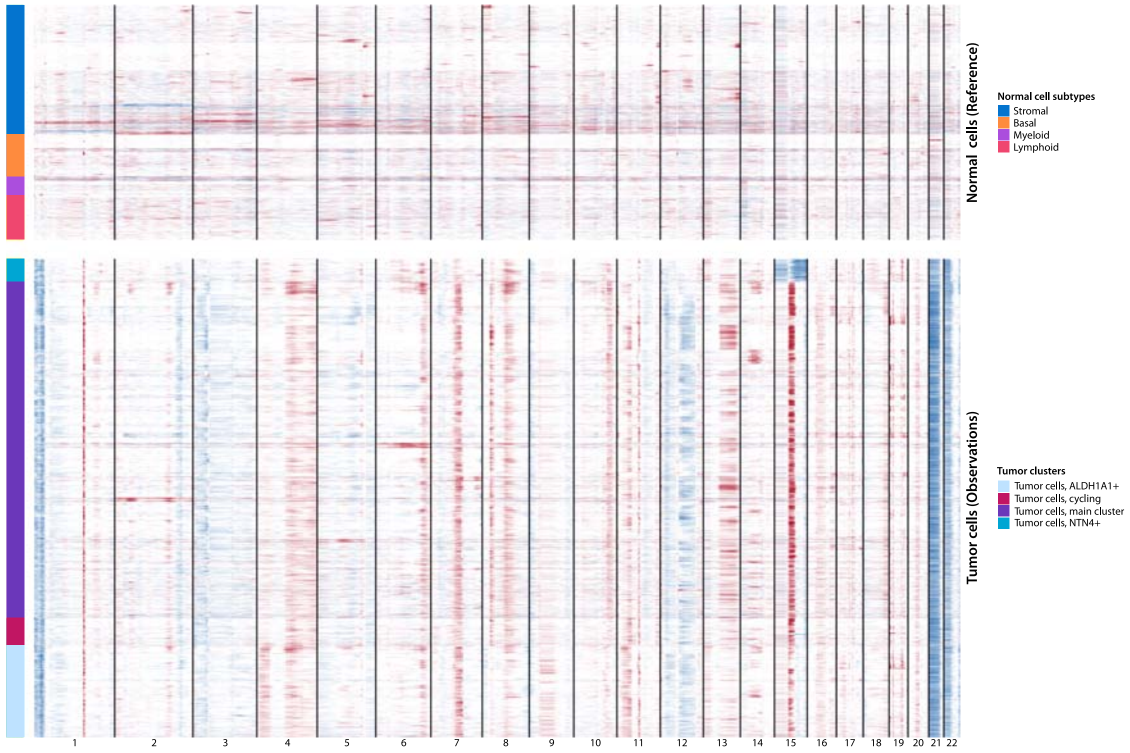

Figure EV2. Heatmap of single-cell copy number profiles inferred from single-nucleus RNA sequencing data.

Notably, loss of chromosome 15 is exclusively found in the NTN4+ tumor cell cluster.

 © The Author(s)

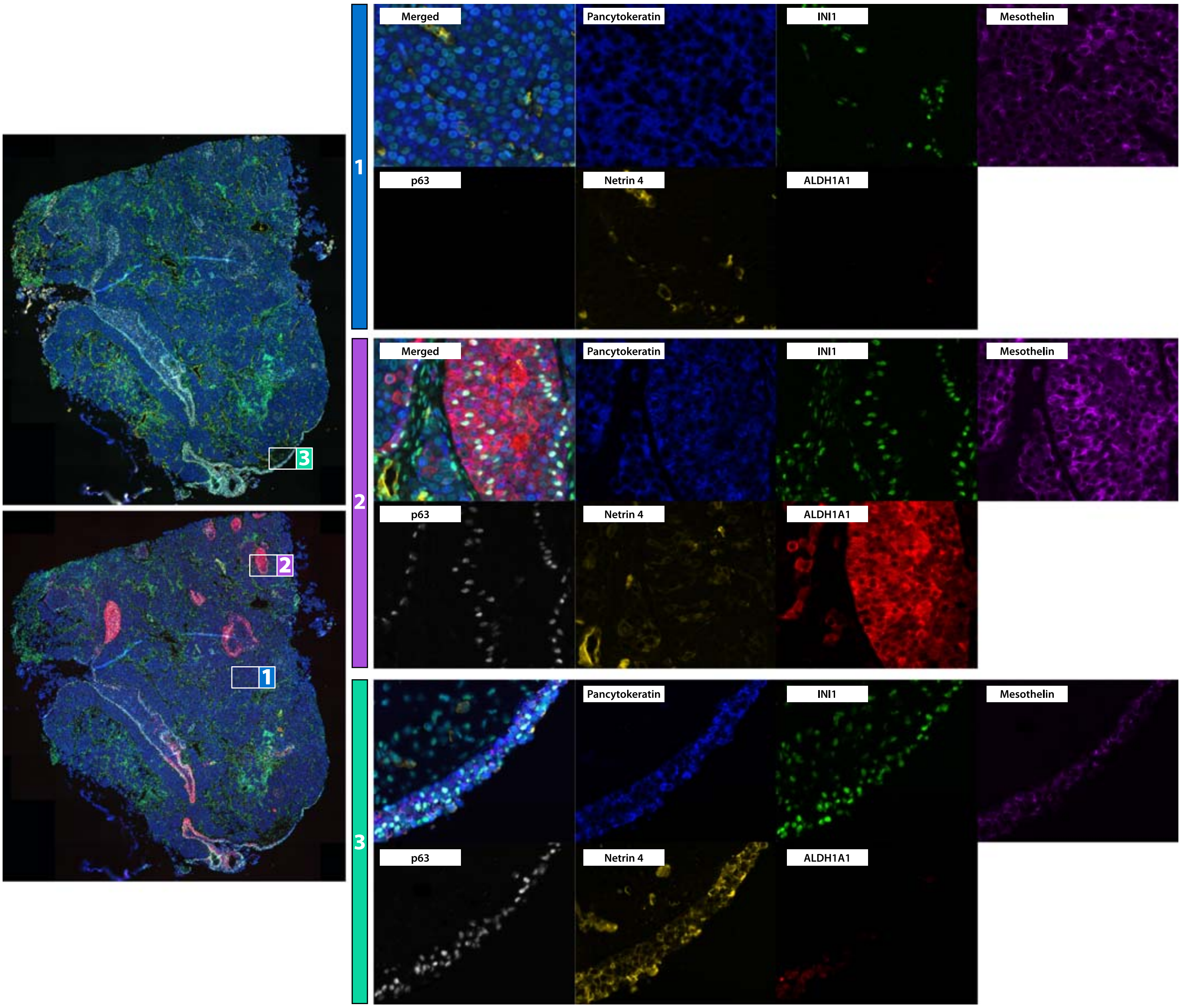

Figure EV3. Validation of tumor niches identified by spatial transcriptomics on the protein level.

Overview and high-magnification sequential immunofluorescence images of a cultured, untreated tissue slice culture recapitulate the three spatial tumor niches defined by spatial transcriptomic analysis. Niche 1 represents the main tumor compartment, characterized by an absence of p63-positive basal cells. NTN4 expression is confined predominantly to endothelial cells lining blood vessels, with only rare expression in tumor cells, and ALDH1A1 is absent. Niche 2 is composed of ALDH1A1-high tumor cells intermingled with p63-positive basal epithelial cells. Netrin 4 again localizes mainly to endothelial cells, retaining nuclear INI1 expression, while tumor cell staining is infrequent. Niche 3 illustrates pagetoid spread of INI1-negative, pancytokeratin-positive tumor cells within the surface epithelium, showing strong Netrin 4 expression and complete absence of ALDH1A1.

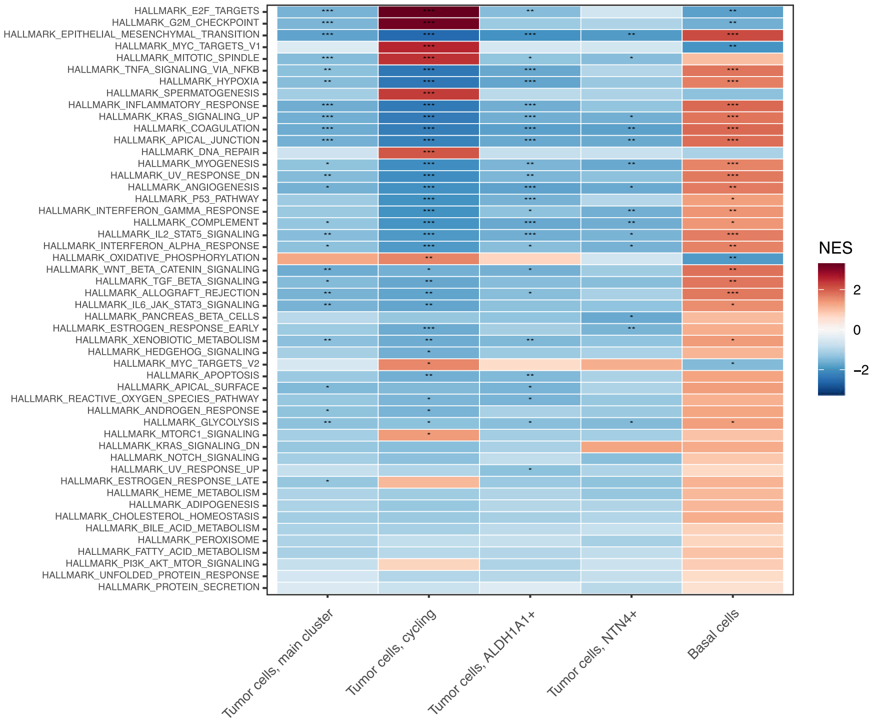

Figure EV4. Heatmap of baseline Hallmark pathway activity score differences between basal and tumor cells in untreated, uncultured control samples.

 © The Author(s)

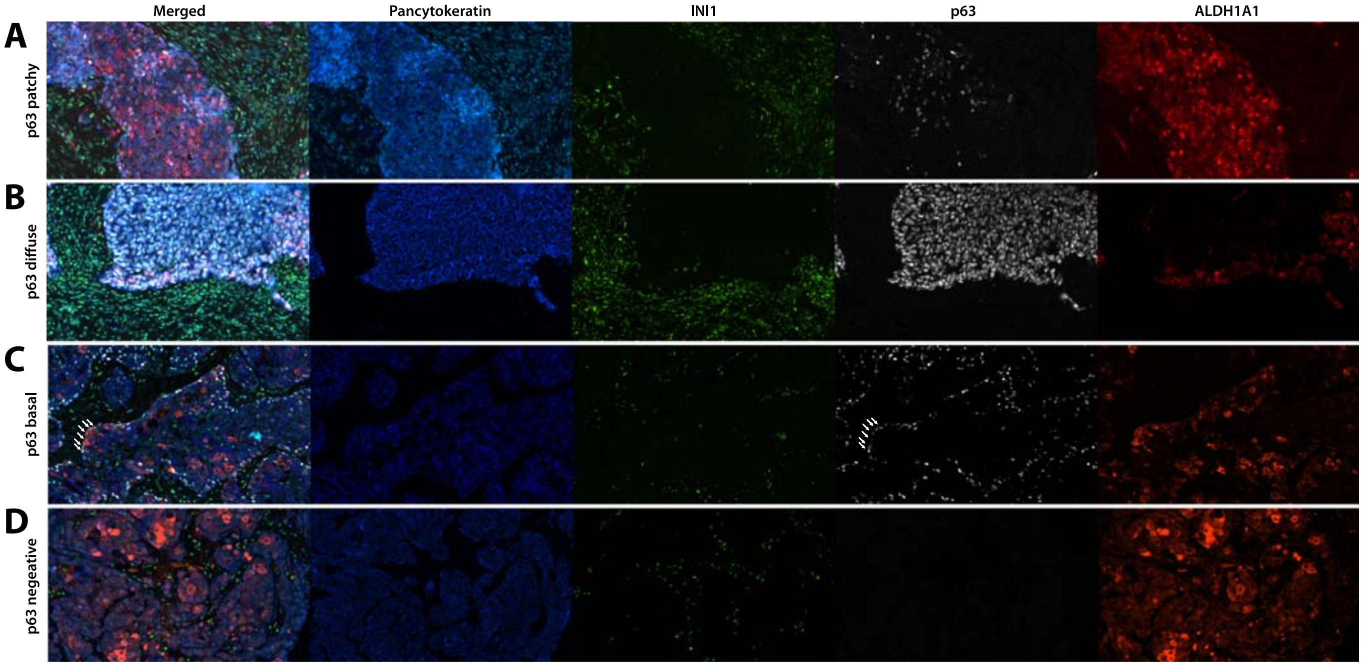

Figure EV5. Representative staining patterns of p63 in the retrospective cohort.

(**A**) Patchy expression of p63 in tumor cells (loss of nuclear INI1). (**B**) Diffuse p63 expression in almost all tumor cells. A similar region of the same sample is also shown in Fig. 4B. (**C**) Predominant expression of p63 in tumor cells at the basal (near stroma; white arrows) layer. (**D**) No p63 expression in tumor cells.
