# Supplementary material for: Spatially resolved ex vivo drug response profiling in SMARCB1-deficient sinonasal carcinoma
Source: EMBO Mol Med. 2026 May 2;18(6):2360–78. doi: 10.1038/s44321-026-00437-1 (PMC13270136; doi:10.1038/s44321-026-00437-1)
Supplement: Supplementary file 6 — Peer Review File [file 44321_2026_437_MOESM6_ESM.pdf]

EMBO
Molecular Medicine

Spatially Resolved Ex Vivo Drug Response Profiling in SMARCB1-Deficient Sinonasal Carcinoma

Philipp Jurmeister, Susanne Flach, Linda Bergmayr, Konstanze Schleich, Edgar Chimal Calderon, Liliana Mochmann, Yauheniya Zhdanovich, Doreen Klingler, Ada Pusztai, Anna Kübler, Christoph Walz, Christoph Benedikt Westphalen, Alexander Beck, Maximilian Leitheiser, Gerben Breimer, Johannes Rijken, Lot Devriese, Philipp Baumeister, Alena Skálová, Simon Schallenberg, Frederick Klauschen, and Andreas Mock

Corresponding author: Philipp Jurmeister (Philipp.jurmeister@med.uni-muenchen.de)

Review Timeline:

Submission Date:	6th Jan 26
Editorial Decision:	10th Feb 26
Revision Received:	31st Mar 26
Editorial Decision:	15th Apr 26
Revision Received:	15th Apr 26
Accepted:	16th Apr 26

Editor: Jingyi Hou

Transaction Report:

(Note: With the exception of the correction of typographical or spelling errors that could be a source of ambiguity, letters and reports are not edited. Depending on transfer agreements, referee reports obtained elsewhere may or may not be included in this compilation. Referee reports are anonymous unless the Referee chooses to sign their reports.)

10th Feb 2026

Dear Prof. Jurmeister,

Thank you again for submitting your work to EMBO Molecular Medicine. We have now received feedback from the two referees who evaluated your manuscript. As you will see in the reports below, both referees express enthusiastic support for your study, while also raising several -relatively minor- issues that will need to be addressed in a revised version of the manuscript.

The referees' recommendations are clear, so I won't repeat the points listed below. It is important to carefully address all the issues raised by the referees. Please feel free to contact me in case you would like to discuss in further detail any of the issues raised by the reviewers.

We would welcome the submission of a revised version within three months for further consideration. As you may already know, our editorial policy allows in principle a single round of major revision, and it is therefore essential to provide responses to the referees' comments that are as complete as possible.

EMBO Molecular Medicine has a "scooping protection" policy, whereby similar findings that are published by others during review or revision are not a criterion for rejection. Should you decide to submit a revised version, I do ask that you get in touch after three months if you have not completed it, to update us on the status.

Please also contact us as soon as possible if similar work is published elsewhere. If other work is published, we may not be able to extend the revision period beyond three months.

Please read below for important editorial formatting and consult our author's guidelines for proper formatting of your revised article for EMBO Molecular Medicine.

I look forward to receiving your revised manuscript.

Sincerely,
Jingyi

Jingyi Hou
Senior Editor
EMBO Molecular Medicine

*****
When preparing your revised manuscript, please refer to our guidelines: https://link.springer.com/journal/44321/submission-guidelines#cms-Revised-submissions. We perform an initial quality control of all revised manuscripts before re-review; failure to include requested items will delay the evaluation of your revision.

We require:

1) A .docx formatted version of the manuscript text (including legends for main figures, EV figures and tables). Please make sure that the changes are highlighted to be clearly visible.

2) Individual production quality figure files as .eps, .tif, .jpg (one file per figure). For guidance, download the 'Figure Guide PDF': https://media.springernature.com/original/springer-cms/rest/v1/content/27825798/data/v1.

3) A .docx formatted letter INCLUDING the reviewers' reports and your detailed point-by-point responses to their comments. As part of the EMBO Press transparent editorial process, the point-by-point response is part of the Review Process File (RPF), which will be published alongside your paper.

4) A complete author checklist, which you can download from our author guidelines. Please insert information in the checklist that is also reflected in the manuscript. The completed author checklist will also be part of the RPF.

5) Please note that all corresponding authors are required to supply an ORCID ID for their name upon submission of a revised manuscript.

6) It is mandatory to include a 'Data Availability' section after the Materials and Methods. Before submitting your revision, primary datasets produced in this study need to be deposited in an appropriate public database, and the accession numbers and database listed under 'Data Availability'. Please remember to provide a reviewer password if the datasets are not yet public.

In case you have no data that requires deposition in a public database, please state so in this section. Note that the Data Availability Section is restricted to new primary data that are part of this study.

7) For data quantification: please specify the name of the statistical test used to generate error bars and P values, the number (n) of independent experiments (specify technical or biological replicates) underlying each data point and the test used to calculate p-values in each figure legend. The figure legends should contain a basic description of n, P and the test applied. Graphs must include a description of the bars and the error bars (s.d., s.e.m.).

8) At EMBO Press we ask authors to provide source data for the main manuscript figures. You will receive a separate email with instructions for providing source data with your revised manuscript, including how to upload and organize the files.

9) Our journal encourages inclusion of *data citations in the reference list* to directly cite datasets that were re-used and obtained from public databases. Data citations in the article text are distinct from normal bibliographical citations and should directly link to the database records from which the data can be accessed. In the main text, data citations are formatted as follows: "Data ref: Smith et al, 2001" or "Data ref: NCBI Sequence Read Archive PRJNA342805, 2017". In the Reference list, data citations must be labeled with "[DATASET]". A data reference must provide the database name, accession number/identifiers and a resolvable link to the landing page from which the data can be accessed at the end of the reference.

10) We replaced Supplementary Information with Expanded View (EV) Figures and Tables that are collapsible/expandable online. A maximum of 5 EV Figures can be typeset. EV Figures should be cited as 'Figure EV1, Figure EV2" etc... in the text and their respective legends should be included in the main text after the legends of regular figures.

- For the figures that you do NOT wish to display as Expanded View figures, they should be bundled together with their legends in a single PDF file called *Appendix*, which should start with a short Table of Content. Appendix figures should be referred to in the main text as: "Appendix Figure S1, Appendix Figure S2" etc.

- Additional Tables/Datasets should be labeled and referred to as Table EV1, Dataset EV1, etc. Legends have to be provided in a separate tab in case of .xls files. Alternatively, the legend can be supplied as a separate text file (README) and zipped together with the Table/Dataset file.

11) The paper explained: EMBO Molecular Medicine articles are accompanied by a summary of the articles to emphasize the major findings in the paper and their medical implications for the non-specialist reader. Please provide a draft summary of your article highlighting

- the medical issue you are addressing,

- the results obtained and

- their clinical impact.

This may be edited to ensure that readers understand the significance and context of the research. Please refer to any of our published articles for an example.

12) Author contributions: You will be asked to provide CRediT (Contributor Role Taxonomy) terms in the submission system. These replace a narrative author contribution section in the manuscript.

13) A Conflict of Interest statement should be provided in the main text.

14) Please provide a 'Synopsis' to further enhance discoverability. Synopses are displayed on the journal webpage and are freely accessible to all readers. They include a short stand first (maximum of 300 characters, including space) as well as 2-5 one-sentences bullet points that summarizes the paper. Please write the bullet points to summarize the key NEW findings. They should be designed to be complementary to the abstract - i.e. not repeat the same text. We encourage inclusion of key acronyms and quantitative information (maximum of 30 words / bullet point). Please use the passive voice. Please attach these in a separate file or send them by email, we will incorporate them accordingly.

Please also provide a visual abstract to illustrate your article as a PNG file 550 px wide x 300-600 px high.

15) Please download and fill our Reagents and Tools Table template (.docx), which you can find in our author guidelines:

https://link.springer.com/journal/44320/submission-guidelines#structuredmethods.

When submitting your revised manuscript, please do not include the Reagents and Tools Table in the Methods section of the manuscript but upload it as a separate file choosing the file type "Reagent Table".

EMBO Molecular Medicine has a "scooping protection" policy, whereby similar findings that are published by others during review or revision are not a criterion for rejection. Should you decide to submit a revised version, I do ask that you get in touch after three months if you have not completed it, to update us on the status.

***** Reviewer's comments *****

Referee #1 (Comments on Novelty/Model System for Author):

This is a high quality well done article with about as much robustness as one can gain from a rare tumor type

Referee #1 (Remarks for Author):

very very well done on an important disease type. there is not that much that is generalizeable, but for an article of this special scope that isOK

Referee #2 (Comments on Novelty/Model System for Author):

I believe this manuscript is novel and highly relevant to the cancer research community, particularly given that SMARCB1-deficient sinonasal carcinoma is a rare malignancy for which very limited molecular data are available, especially using contemporary single-cell and spatial technologies. The study is therefore well suited for publication in EMBO Molecular Medicine.

The authors employ appropriate and well-established methodologies to interrogate patient-derived biopsy material using complementary experimental and computational approaches. However, in its current form, the manuscript would benefit from the inclusion of additional data that are likely already available to the authors, as well as from further analyses that should be feasible using the existing datasets. These additions would substantially strengthen the biological interpretation and the overall impact of the study.

Referee #2 (Remarks for Author):

Using recent advances in sequencing technologies, Jurmeister et al. address tumour heterogeneity and drug responses in SMARCB1-deficient sinonasal carcinoma (SDSC). The topic of this manuscript is both important and novel, and the study provides new insights into the biology and potential treatment of this rare malignancy. In particular, the work offers valuable information on tumour heterogeneity and tumour origins.
The authors skilfully use a single biopsy to generate a comprehensive dataset comprising genomic profiling, H&E staining, and ex vivo drug testing using tissue-slice cultures, followed by snRNA-seq and sequential immunofluorescence guided by spatial transcriptomics performed on a naïve sample. The analysis identifies four tumour subclusters (the main tumour cluster, cycling tumour cells, an ALDH1A1-positive subcluster and an NTN4-positive subcluster) and maps their spatial localisation to distinct tumour niches.
Overall, the authors present a unique and valuable dataset for a rare cancer entity for which in vitro models are largely lacking, making this study a potentially important resource for the SDSC community and beyond. However, in order for the scientific community to fully benefit from this resource, several aspects of the analysis, data presentation and clarity should be improved. Importantly, the following points can be addressed using the existing data and do not require additional experiments.
1. The analysis identifies four tumour subpopulations and a basal epithelial population. The finding that the basal cells are deletion-free and retain SMARCB1 expression, in contrast to the tumour cells, is particularly intriguing given that the basal layer represents the stem and progenitor compartment responsible for epithelial maintenance. A discussion of this observation could provide important insight into the cellular origin of SDSC and potentially other epithelial tumours.
Specifically, the authors should discuss which epithelial cell type is most likely to give rise to SDSC and how the basal population identified here relates to the tumour populations. To further support this, I suggest re-clustering the epithelial compartment only (the four tumour clusters together with the basal cluster) and performing pseudotime or trajectory analysis. In addition, visualisation of a broader panel of epithelial markers (e.g. keratins and mucins) across tumour and basal clusters (for example using dot plots) would be informative.
2. Trajectory analysis may also clarify the nature of the NTN4-positive subcluster. Based on Supplementary Fig. S5, this population appears to acquire a novel copy number alteration on chromosome 15, suggesting clonal evolution rather than purely functional divergence. The authors should comment on whether this subcluster represents a genetically distinct tumour clone and how this relates to their biological interpretation.

The ALDH1A1-positive population is of particular interest, as it displays features consistent with cancer stem-like cells. The authors provide convincing evidence for the presence of ALDH1A1-positive cells in SDSC and validate this finding in an independent cohort, showing increased ALDH1A1 expression in two recurrent tumours compared with primary tumours. They further show that this population is selectively targeted by the mTOR inhibitor sapanisertib. However, the evidence supporting this conclusion remains incomplete.

3. The conclusions regarding sapanisertib should be more cautious, as drug effects are demonstrated using only a single index sample. Moreover, the spatial distribution of ALDH1A1-positive cells varies across additional SDSC specimens and does not appear to be consistently associated with the basal compartment, suggesting that drug sensitivity may vary between tumours. The authors should clarify whether the H&E and immunofluorescence analyses after drug treatment were performed on one or multiple tissue slices and, if more than one slice was analysed, whether these were derived from independently treated samples. This information is currently unclear.

4. To support the proposed cluster-specific action of sapanisertib, enrichment of the PI3K-AKT-mTOR pathway is discussed. However, the underlying data are not shown, making it difficult to assess the specificity of this effect. Pathway enrichment should be presented across all epithelial clusters, including the basal and NTN4-positive populations. In addition, it would be important to indicate how these enrichments change relative to untreated samples.

In addition, is the PI3K-AKT-mTOR pathway the top enriched pathway in the ALDH1A1-positive subcluster? If not, what other signalling pathways are most prominent in this population?

5. It would be valuable to compare the identified epithelial populations with recently described cell types in normal nasal and sinonasal epithelium (i.e. Reddy KD et al., Nat Commun 2025, 16(1):9494; Ruysseveldt E et al., I Allergy Clin Immunol 2024, 154(3): 631-643; Liao G et al., Immunity 2025, 58(10):2593-2608). Such a comparison would be highly relevant for interpreting the potential dual origins of epithelial cells (basal versus tumour populations) in this study.

6. The current marker lists for the tumour clusters are restricted to the top 50 genes per cluster. For completeness, the authors should provide the full lists of significant markers. Importantly, marker genes for the basal cluster should also be reported.

7. A complete list of detected mutations should be provided. In addition, the biological and clinical relevance of high MSLN expression should be discussed.

8. Although mesothelin staining is mentioned (p. 12, paragraph beginning with "To validate the effect of sapanisertib"), the corresponding images are not shown. It would be informative to assess whether mesothelin expression overlaps with ALDH1A1 staining.

9. Similarly, p63 and NTN4 staining is not shown in the final section. The authors state that none of the external samples displayed intermingling of p63-positive normal basal cells. It is unclear whether this means that p63 staining was absent or that the staining pattern differed from that observed in the index case. This point is important for understanding the potential presence of a normal basal epithelial population that may support tumour stem-like cells. As these stainings appear to have been performed, they should be shown in a supplementary figure.

10. In the Discussion, the term "2D cell culture organoids" is used. The authors presumably mean "3D" organoid cultures.

11. The methods corresponding to Fig. 1A-B (H&E, immunocytochemistry and MRI) should be described.

12. When relevant, software versions should be reported, and a link to the PathInfony pipeline should be provided. Appropriate references for the PROGENy and CollecTRI networks should also be included.

13. The quality of the material for snRNA-seq is currently assessed using viable cell counts. This should be replaced with metrics appropriate for nuclei-based assays, such as nuclei integrity and nuclei counts.

14. In-text references to Figure 3 should be corrected.

15. Relevant abbreviations should be defined when first introduced, and gene name formatting (italicisation) should follow standard guidelines consistently throughout the manuscript.

Point-by-point response

Reviewer comments are displayed in blue.
Author replies are shown in black.
Citations from the revised main manuscript are written in italics.

Reviewer #1

Comment 1: "This is a high quality well done article with about as much robustness as one can gain from a rare tumor type

very very well done on an important disease type. there is not that much that is generalizeable, but for an article of this special scope that is OK"

Reply: We sincerely thank the reviewer for the very positive and encouraging evaluation of our work and for recognizing its robustness despite the rarity of this tumor entity.

Reviewer #2

Comment 1: "The analysis identifies four tumor subpopulations and a basal epithelial population. The finding that the basal cells are deletion-free and retain SMARCB1 expression, in contrast to the tumor cells, is particularly intriguing given that the basal layer represents the stem and progenitor compartment responsible for epithelial maintenance. A discussion of this observation could provide important insight into the cellular origin of SDSC and potentially other epithelial tumors.
Specifically, the authors should discuss which epithelial cell type is most likely to give rise to SDSC and how the basal population identified here relates to the tumor populations. To further support this, I suggest re-clustering the epithelial compartment only (the four tumor clusters together with the basal cluster) and performing pseudotime or trajectory analysis. In addition, visualisation of a broader panel of epithelial markers (e.g. keratins and mucins) across tumor and basal clusters (for example using dot plots) would be informative. Trajectory analysis may also clarify the nature of the NTN4-positive subcluster. Based on Supplementary Fig. S5, this population appears to acquire a novel copy number alteration on chromosome 15, suggesting clonal evolution rather than purely functional divergence. The authors should comment on whether this subcluster represents a genetically distinct tumor clone and how this relates to their biological interpretation."

Reply: We thank the reviewer for highlighting this interesting and biologically highly relevant aspect. This point overlaps in part with our response to Comment 4, as both comments address the relationship between non-neoplastic epithelial cells and the tumor cell populations, as well as the possible cell of origin of SDSC.

In response to the reviewer's suggestion, we first performed differential expression analysis across basal and tumor cell populations and focussed further comparisons on keratins, mucins, and cell surface markers (new **Appendix Figure S5**). As expected, the basal compartment showed strong enrichment for canonical basal markers, including *TP63*, *KRT5*, and *COL17A1*, whereas these markers were reduced in the tumor cell clusters. NTN4+ and ALDH1A1+ tumor cells both showed high expression of *CEACAM6*, but otherwise displayed distinct epithelial programs: the NTN4+ subcluster was characterized by elevated *CD70*, whereas the ALDH1A1+ subcluster showed increased expression of *CD38*, *MUC4*, and *MUC15*. To interpret these divergent differentiation-associated programs in the context of normal sinonasal epithelium, we applied gene-signature scoring using UCell and compared the tumor subclusters to the publicly available single-cell reference dataset of normal sinonasal epithelial cell types by Liao et al., suggested by the reviewer in Comment 4. In this analysis, main-cluster tumor cells showed the greatest similarity to basal epithelial signatures, whereas ALDH1A1+ tumor cells aligned most closely with serous/secretory signatures and NTN4+ tumor cells with intermediate ciliated signatures. Taken together, these findings suggest that the tumor compartment spans a broad spectrum of epithelial differentiation states. This could be compatible with the hypothesis that SMARCB1-deficient tumor cells arise from a progenitor-like epithelial population with basal characteristics that is still capable of giving rise to tumor cells showing subtle properties of several sinonasal epithelial cell types.

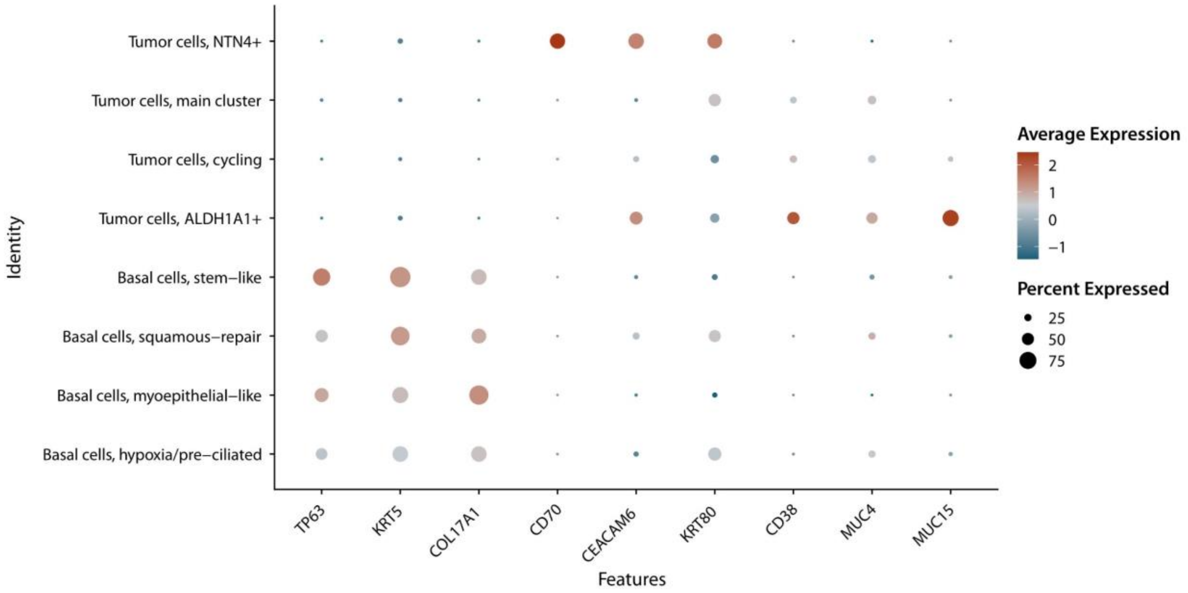

New Appendix Figure S5 Dotplot showing the expression of selected keratins, mucins and cell surface markers between different tumor and basal cell clusters.

During the integration of our data with the single-cell reference dataset of normal sinonasal epithelial cell types by Liao et al., we observed that the basal population from our dataset was not homogeneous, but instead contained several distinct subpopulations. Thus, we decided to perform subclustering of the basal compartment and identified four different cell states: (i) a stem-like basal cluster enriched for *LGR5* and *LGR6*, (ii) a squamous-like basal cluster enriched for *KRT6A* and *SPRR1B*, (iii) a hypoxic basal cluster characterized by *CA9*, *EGLN3*, and *VEGFA*, and (iv) a myoepithelial-like basal cluster with elevated *SOX10*,

KRT14, *MYLK*, and *ACTA2* expression (new panel for **Figure 1H** and new **Appendix Figure S6**). These findings indicate that our non-neoplastic basal cell cluster itself contains substantial phenotypic diversity and likely includes both stem/progenitor-like and more differentiated basal cell states.

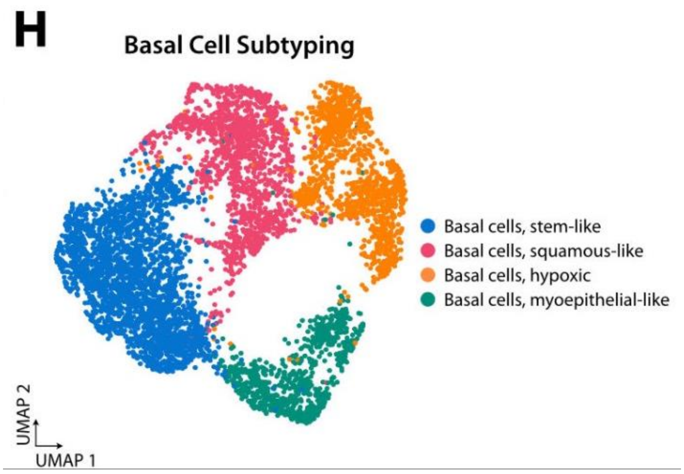

New Figure 1H UMAP visualization of basal-cell subclustering revealed four distinct basal populations with stem-like, squamous-like, hypoxic, and myoepithelial-like features.

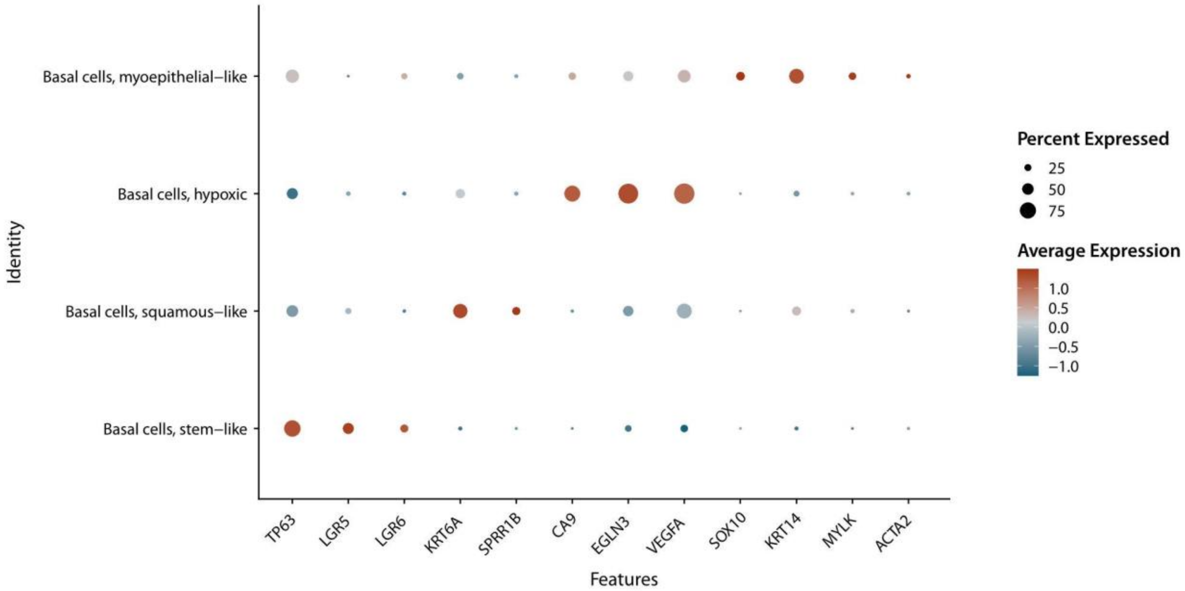

New Appendix Figure S6 Dotplot showing the expression of characteristic markers genes of the different basal cell subclusters.

We then performed trajectory inference within the epithelial compartment (containing both basal and tumor cells) using Slingshot, selecting the stem-like basal cluster as the root because it showed the strongest stem/progenitor-associated expression profile (new figure panel **Figure 1I**). This analysis identified three trajectories in the epithelial compartment. One trajectory connected the stem-like basal cells to the main tumor cluster and then progressed through cycling tumor cells toward the ALDH1A1+ tumor cell cluster, while a second tumor-associated trajectory also passed through the main tumor cluster but diverged toward the NTN4+ tumor cell cluster. A third trajectory remained within the non-malignant

basal epithelial compartment and extended from stem-like basal cells through squamous-like and hypoxic basal states toward the myoepithelial-like basal cluster. These results - in combination with the diverging expression profiles of the tumor cell clusters described above - support a model in which the stem-like basal cell population represents the closest non-neoplastic counterpart (potentially the cell of origin) of tumor cells and in which the ALDH1A1+ and NTN4+ tumor cell clusters correspond to distinct tumor differentiation states (or even distinct clones) emerging along separate branches of tumor evolution. As outlined by the reviewer, this interpretation could further be supported by the unique loss of chromosome 15, only observed in NTN4+ tumor cells.

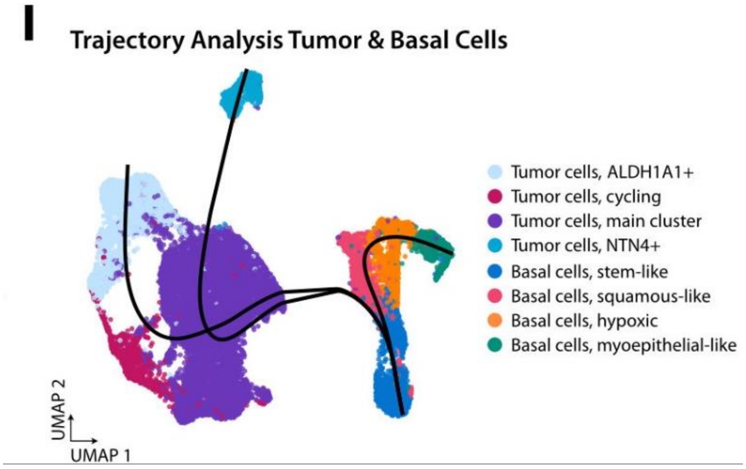

New Figure 1I UMAP with Slingshot-based pseudotime/trajectory inference identified three lineages. One lineage connected stem-like basal cells to the main tumor cluster and progressed via cycling tumor cells toward the ALDH1A1+ tumor subcluster. A second tumor-associated lineage also passed through the main tumor cluster but diverged toward the NTN4+ tumor subcluster. A third lineage remained confined to the non-malignant basal epithelial compartment.

We updated the Results section accordingly:

"We further examined the expression of a broader panel of keratins, mucins, and cell-surface markers and identified clear differences between the tumor subclusters. NTN4+ and ALDH1A1+ tumor cells both showed high expression of CEACAM6, but otherwise displayed distinct epithelial programs: the NTN4+ subcluster was characterized by elevated CD70, whereas the ALDH1A1+ subcluster showed increased expression of CD38, MUC4, and MUC15 (Appendix Figure S5). To interpret these divergent differentiation-associated programs in the context of normal sinonasal epithelium, we applied gene-signature scoring and compared the tumor subclusters to a publicly available single-cell reference dataset of normal sinonasal epithelial cell types.18 In this analysis, main-cluster tumor cells showed the greatest similarity to basal cell signatures, whereas ALDH1A1+ tumor cells aligned most closely with serous cell signatures and NTN4+ tumor cells with intermediate ciliated signatures (Figure 1F).

(...)

Further subclustering of the basal cell cluster revealed four distinct cell states (Figure 1H): (i) a stem-like basal cluster enriched for LGR5 and LGR6, (ii) a squamous-like basal cluster enriched for KRT6A and SPRR1B, (iii) a hypoxic basal cluster characterized by CA9,

EGLN3, VEGFA, and (iv) a myoepithelial-like basal cluster with elevated SOX10, KRT14, MYLK, and ACTA2 expression (Appendix Figure S6).

We then performed trajectory inference within the epithelial compartment (containing both basal and tumor cells) using Slingshot, selecting the stem-like basal cluster as the root because it showed the strongest stem/progenitor-associated expression profile. This analysis identified three trajectories in the epithelial compartment. One trajectory connected the stem-like basal cells to the main tumor cluster and then progressed through cycling tumor cells toward the ALDH1A1+ tumor cell cluster, while a second tumor-associated trajectory also passed through the main tumor cluster but diverged toward the NTN4+ tumor cell cluster. A third trajectory remained within the non-malignant basal epithelial compartment and extended from stem-like basal cells through squamous-like and hypoxic basal states toward the myoepithelial-like basal cluster."

Furthermore, the revised Discussion now reads:

"Comparison with a normal sinonasal epithelial reference further supported a broad differentiation spectrum, with each tumor cell cluster showing greatest transcriptional similarity to distinct normal epithelial programs. Together, these findings are compatible with the hypothesis that SDSC arises in a progenitor-like epithelial context with basal characteristics and retains sufficient plasticity to adopt differentiation-associated features of multiple sinonasal epithelial lineages. In our dataset, the stem-like basal cell cluster most closely matched such a progenitor-like state, and trajectory inference supported a branching organization in which ALDH1A1+ and NTN4+ tumor cells occupy divergent endpoints, consistent with distinct differentiation-associated programs. This interpretation is further supported by inferred copy-number profiles indicating a chromosome 15 loss uniquely enriched in the NTN4+ subcluster, suggestive of subclonal evolution. While pseudotime/trajectory analyses provide computational ordering of transcriptional states rather than definitive lineage directionality, these results motivate a model in which stem-like basal cells represent a plausible cell of origin for SDSC with subsequent diversification."

Comment 2: "The conclusions regarding sapanisertib should be more cautious, as drug effects are demonstrated using only a single index sample. Moreover, the spatial distribution of ALDH1A1-positive cells varies across additional SDSC specimens and does not appear to be consistently associated with the basal compartment, suggesting that drug sensitivity may vary between tumors.
The authors should clarify whether the H&E and immunofluorescence analyses after drug treatment were performed on one or multiple tissue slices and, if more than one slice was analysed, whether these were derived from independently treated samples. This information is currently unclear."

Reply: We agree with the reviewer that conclusions regarding Sapanisertib must be interpreted with appropriate caution. Accordingly, we have further revised the Abstract, Results, and Discussion to emphasize the exploratory, hypothesis-generating nature of these findings and the fact that the results have been derived from a single index case. The table below includes a comparison of revised statements:

Old version	Revised version
“Ex vivo TSC drug testing revealed striking selective vulnerability”	*“Ex vivo TSC drug testing revealed a striking response in the index tumor”*
“By uncovering a stem-like, ALDH1A1+ niche potentially vulnerable to mTOR inhibition (…)”	*“By uncovering a stem-like, ALDH1A1+ niche potentially vulnerable to mTOR inhibition (...)”*
“Collectively, these findings nominate mTOR inhibition as a rational strategy to weaken or eradicate a putative recurrence-founder population in SDSC.“	*“Collectively, these findings support mTOR pathway inhibition as a candidate vulnerability for targeting recurrence-founder population in SDSC that warrants validation in additional cases.”*
“Given the lethality of SDSCs and the scarcity of models, these findings - while early - provide a concrete biological framework and actionable signals to prioritize in translational studies and early-phase clinical trials.“	*“Given the lethality of SDSCs and the scarcity of models, these findings - while early - provide a concrete biological framework and actionable signals to prioritize in future preclinical studies.”*
“Functionally, the TSC drug screen uncovered a striking selective vulnerability to the mTOR kinase inhibitor Sapanisertib”	*“Functionally, the TSC drug screen indicated a potential selective vulnerability to the mTOR kinase inhibitor Sapanisertib”*
NA	*The pronounced response to Sapanisertib and the observed transcriptional changes following treatment are consistent with involvement of the mTOR/PI3K axis, although we cannot exclude additional indirect or off-target contributions to the observed effects.*
“Our data suggests that targeting mTOR signaling can directly debulk this compartment within its native niche.”	*Our data suggests that targeting mTOR signaling can debulk this compartment in the index case TSC model within its native niche.*

We agree that it is also important to emphasize that the distinctive spatial pattern observed in the index case (non-malignant basal cells intermingled among ALDH1A1+ tumor cells) was not observed in the retrospective case series, and to discuss the potential implications of this difference. We have therefore added the following paragraph to the revised Discussion section:

“In particular, it remains unclear whether the spatial organization with non-malignant basal cells intermingled with ALDH1A1+ tumor cells observed in the index case is essential for the proposed sensitivity to Sapanisertib. As this specific pattern was not consistently observed in other tumors from the retrospective cohort, their response to treatment may, in principle, differ.”

Furthermore, we apologize that the strategy for generating FFPE sections for the different analyses was not clearly communicated in the original manuscript. We have now clarified in the Methods section that, after fixation and embedding, each treatment condition was sectioned to generate an H&E-stained slide, which was reviewed by a board-certified pathologist to confirm the presence of representative tumor tissue. Due to limited tissue availability, one additional blank section per condition was reserved for potential sequential immunofluorescence (seqIF). The remaining material from this block, together with all tissue from a second block processed under the same condition, was pooled to obtain sufficient material for snRNAseq. This strategy was chosen because true replication for snRNAseq

was not feasible given the very limited amount of tissue, and because we intended to use seqIF primarily as a protein-level validation of the snRNAseq findings rather than as an independent replicate experiment.

We now describe this workflow explicitly in the revised Methods section:

"After fixation and embedding, samples were sectioned to generate an H&E-stained slide. All stained sections were reviewed by a board-certified pathologist, confirming the presence of representative tumor tissue in each sample. Owing to limited tissue availability, one FFPE block was used to generate one additional section reserved for potential sequential immunofluorescence (seqIF). The remaining material from this block, together with all tissue from the second block processed under the same condition, was pooled to obtain sufficient nuclei/cell numbers for single nucleus RNA sequencing (snRNAseq) analysis.
Uncultured, untreated control specimens yielded more material and - in addition to H&E sections - were used to generate additional sections for Xenium spatial transcriptomics as well as multiple blank slides reserved for seqIF."

Comment 3: "To support the proposed cluster-specific action of sapanisertib, enrichment of the PI3K-AKT-mTOR pathway is discussed. However, the underlying data are not shown, making it difficult to assess the specificity of this effect. Pathway enrichment should be presented across all epithelial clusters, including the basal and NTN4-positive populations. In addition, it would be important to indicate how these enrichments change relative to untreated samples.
In addition, is the PI3K-AKT-mTOR pathway the top enriched pathway in the ALDH1A1-positive subcluster? If not, what other signalling pathways are most prominent in this population?"

Reply: We agree with the reviewer that this section was overly superficial and could easily be misunderstood. The reported "upregulation" refers to changes in inferred pathway activity in Sapanisertib-treated samples, but this was not communicated transparently in the original manuscript. We have therefore redone this analysis from scratch, following the reviewer's suggestions.

To investigate baseline pathway activities, we first examined the cultured but untreated control samples (**new Figure EV4**). A global comparison of pathway activity across the different tumor cell subclusters and basal cells revealed only minor differences between tumor clusters, with the expected exception of proliferation-related pathways enriched in cycling tumor cells. Notably, no consistent baseline enrichment of mTOR/PI3K-related pathway activity was observed in ALDH1A1+ or NTN4+ tumor cells.

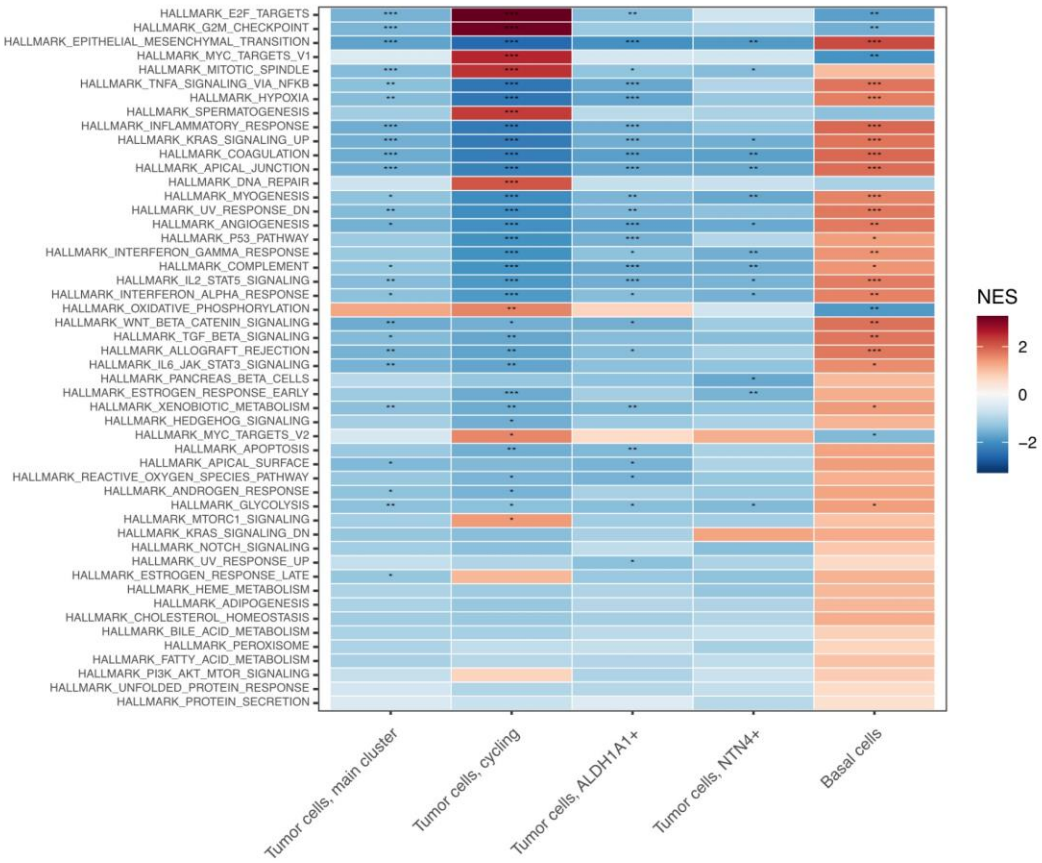

New Figure EV4 Heatmap of baseline Hallmark pathway activity score differences between basal and tumor cells in untreated, uncultured control samples.

Next, we compared pathway activity changes across tumor cell subclusters under different treatment conditions. While pathway activities in the main cluster, cycling, and NTN4+ tumor cells were relatively stable across treatments, ALDH1A1+ tumor cells showed markedly altered pathway activity scores under Sapanisertib treatment compared with both the cultured control and all other tumor cell subclusters. Among the ten most strongly upregulated pathways were p53 and reactive oxygen species pathways, consistent with induction of cellular stress responses and programmed cell death. Furthermore, strong upregulation of unfolded protein response and protein secretion suggested severe ER/proteostasis stress, consistent with the known effect of mTORC1/2 inhibition in disrupting protein homeostasis. As described before, mTOR/PI3K gene sets were also among the most elevated pathways, potentially reflecting compensatory feedback mechanisms. For example, S6K1-dependent inhibitory phosphorylation of IRS-1 has been reported to be induced upon mTOR inhibition, which can result in reactivation of upstream PI3K/AKT signaling.

Among the most strongly downregulated pathways were early and late estrogen response, suggesting suppression of a pro-survival signaling axis in this subcluster. NOTCH signaling was also reduced in sapanisertib-treated ALDH1A1+ cells. NOTCH is a conserved regulator of stemness and self-renewal programs in multiple cancers. Thus, its downregulation in the ALDH1A1+ subpopulation may indicate disruption of stem-like properties.

Taken together, the pathway changes observed in ALDH1A1+ tumor cells under sapanisertib treatment are consistent with a model of mTOR inhibition-associated proteotoxic/ER stress, activation of p53/apoptosis programs, and concurrent attenuation of pro-survival and stemness-associated signaling, selectively affecting this subcluster.

Since NTN4+ tumor cells were virtually absent in the sapanisertib-treated samples, effects on pathway activity in this subcluster could not be assessed.

The updated Results section now reads:

"To gain mechanistic insight into the selective loss of ALDH1A1+ tumor cells following Sapanisertib treatment, we performed single-cell pathway activity analysis. In untreated control samples, baseline pathway activity patterns were broadly similar across tumor subclusters and basal cells, with expected enrichment of cell-cycle-associated programs in cycling tumor cells (Figure EV4). In particular, we did not observe consistent baseline enrichment of mTOR/PI3K pathway activity in ALDH1A1+ or NTN4+ tumor cells.

In contrast, under Sapanisertib treatment, ALDH1A1+ tumor cells showed the most pronounced and selective changes in pathway activity compared with both the cultured control and the other tumor subclusters (Figure 3H and Appendix Figure S9). Upregulated programs included apoptosis/p53-associated responses and oxidative stress signatures, together with strong induction of unfolded protein response/protein handling pathways consistent with ER/proteostasis stress. At the same time, pathway activity signatures annotated to mTOR/PI3K signaling were increased, consistent with a potential feedback response to mTOR pathway inhibition. Conversely, estrogen-response-associated programs and NOTCH signaling activity were reduced in Sapanisertib-treated ALDH1A1+ cells."

Furthermore, we have revised the Discussion section:

"While baseline pathway activity profiling in untreated controls did not reveal prominent enrichment of mTOR/PI3K-related pathways in ALDH1A1+ or NTN4+ tumor cells relative to other malignant subclusters, striking differences emerged upon treatment. ALDH1A1+ tumor cells showed the most pronounced transcriptional response to Sapanisertib, which were consistent with proteotoxic stress, a well known effect of mTOR inhibition. Concurrent suppression of pro-survival signaling and reduced NOTCH pathway activity further support disruption of stemness-associated programs in this subpopulation. Notably, mTOR/PI3K gene sets were among the most elevated pathways after treatment, a pattern that may reflect compensatory feedback within the PI3K/AKT/mTOR axis. For example, S6K1-dependent inhibitory phosphorylation of IRS-1 has been reported to be induced upon mTOR inhibition, which can result in reactivation of upstream PI3K/AKT signaling.

Together, these data suggest that the ALDH1A1+ compartment occupies a therapeutically vulnerable niche and is closely spatially associated with basal cell and vascular compartments. The pronounced response to Sapanisertib and the observed transcriptional changes following treatment are consistent with involvement of the mTOR/PI3K axis, although we cannot exclude additional indirect or off-target contributions to the observed effects."

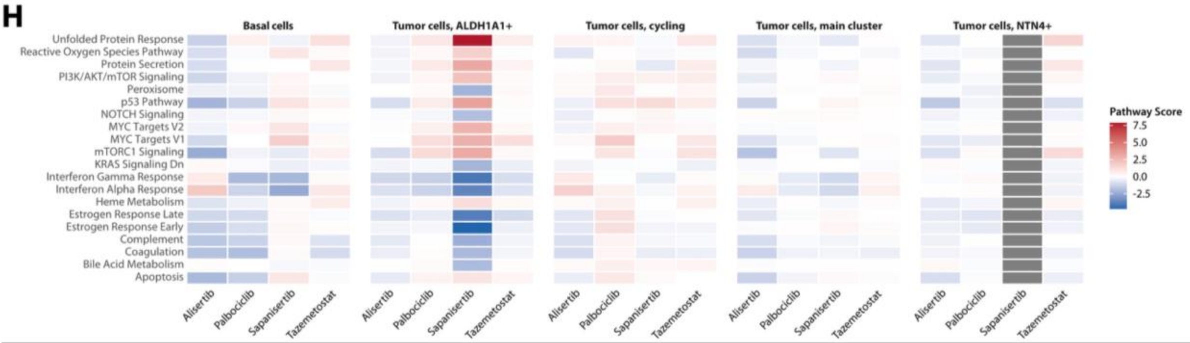

New Figure 3H Heatmap showing pathway activity changes for the top 10 upregulated and top 10 downregulated Hallmark pathways identified in ALDH1A1+ tumor cells treated with Sapanisertib. Values for Sapanisertib treatment effects in NTN4+ tumor cells could not be calculated since they were no longer present after treatment.

Comment 4: "It would be valuable to compare the identified epithelial populations with recently described cell types in normal nasal and sinonasal epithelium (i.e. Reddy KD et al., Nat Commun 2025, 16(1):9494; Ruysseveldt E et al., I Allergy Clin Immunol 2024, 154(3): 631-643; Liao G et al., Immunity 2025, 58(10):2593-2608). Such a comparison would be highly relevant for interpreting the potential dual origins of epithelial cells (basal versus tumor populations) in this study."

Reply: We thank the reviewer for this interesting suggestion. We used the published single-cell reference dataset of normal sinonasal epithelium by Liao et al. to further contextualize the differentiation states of our tumor and basal cell populations. As described above, this analysis enabled a more detailed assessment of differentiation-associated differences among the tumor subclusters and additionally revealed heterogeneity within the non-malignant basal cell compartment. These results have already been described in detail in our response to Comment 1.

Comment 5: "The current marker lists for the tumor clusters are restricted to the top 50 genes per cluster. For completeness, the authors should provide the full lists of significant markers. Importantly, marker genes for the basal cluster should also be reported."

Reply: We thank the reviewer for this suggestion. We have now provided the complete output of differential expression analysis for all marker genes for each cell type in the updated Table EV3.

Comment 6: "A complete list of detected mutations should be provided. In addition, the biological and clinical relevance of high MSLN expression should be discussed."

Reply: We thank the reviewer for this valuable suggestion. We have added a comprehensive list of detected mutations as a new Table EV2. In addition, we have expanded the Discussion to address the biological and clinical relevance of high mesothelin expression. The updated Discussion now reads:

“Mesothelin has several established oncogenic functions: it enhances proliferation and survival by activating PI3K and AKT signaling, promotes invasion via MMP-9 upregulation, and polarizes tumor-associated macrophages toward an immunosuppressive M2-like phenotype. Interestingly, Mesothelin expression in normal tissues is restricted to mesothelial linings.”

Comment 7: “Although mesothelin staining is mentioned (p. 12, paragraph beginning with "To validate the effect of sapanisertib"), the corresponding images are not shown. It would be informative to assess whether mesothelin expression overlaps with ALDH1A1 staining.”

Reply: We thank the reviewer for pointing out that the mesothelin immunostaining was mentioned in the text but not shown in the submitted figures. We have now added corresponding panels to the revised **Figure EV3**, enabling visual assessment of mesothelin expression in relation to the different tumor compartments. In addition, we added a new **Appendix Figure S4** showing mesothelin staining in an untreated TSC control, illustrating tumor-cell-specific expression. Finally, mesothelin expression in the independent validation cohort remains shown in Figure 4E, as in the original submission.

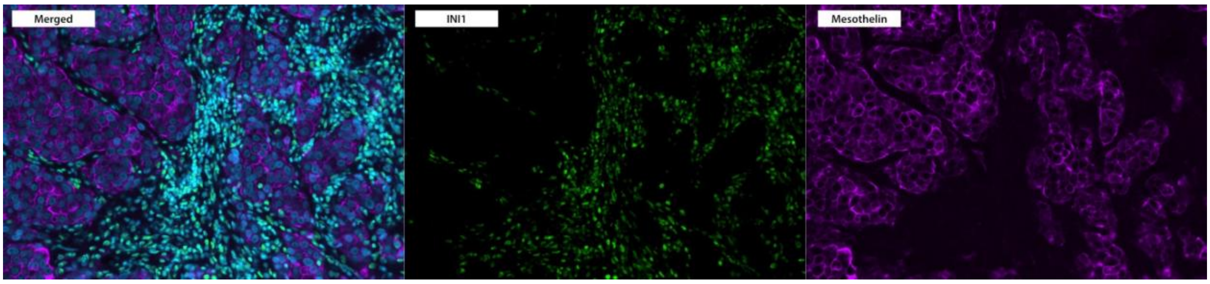

New Appendix Figure S4 Representative sequential immunofluorescence images of an uncultured, untreated control specimen from the index case. Consistent with the single-nucleus RNA sequencing findings, tumor cells (identified by loss of nuclear INI1) showed strong mesothelin protein expression, whereas stromal fibroblasts and immune cells with retained nuclear INI1 showed no detectable mesothelin staining.

Regarding the reviewer’s suggestion to assess overlap between mesothelin and ALDH1A1, we note that *MSLN* expression was a shared feature across all tumor cell subclusters (see also Figure 1E) and was not restricted to a specific transcriptional subcluster or spatial tumor compartment. This is now also evident at the protein level in the newly added panels in **Figure EV3**, where the relationship of mesothelin staining to the tumor compartment can be directly appreciated.

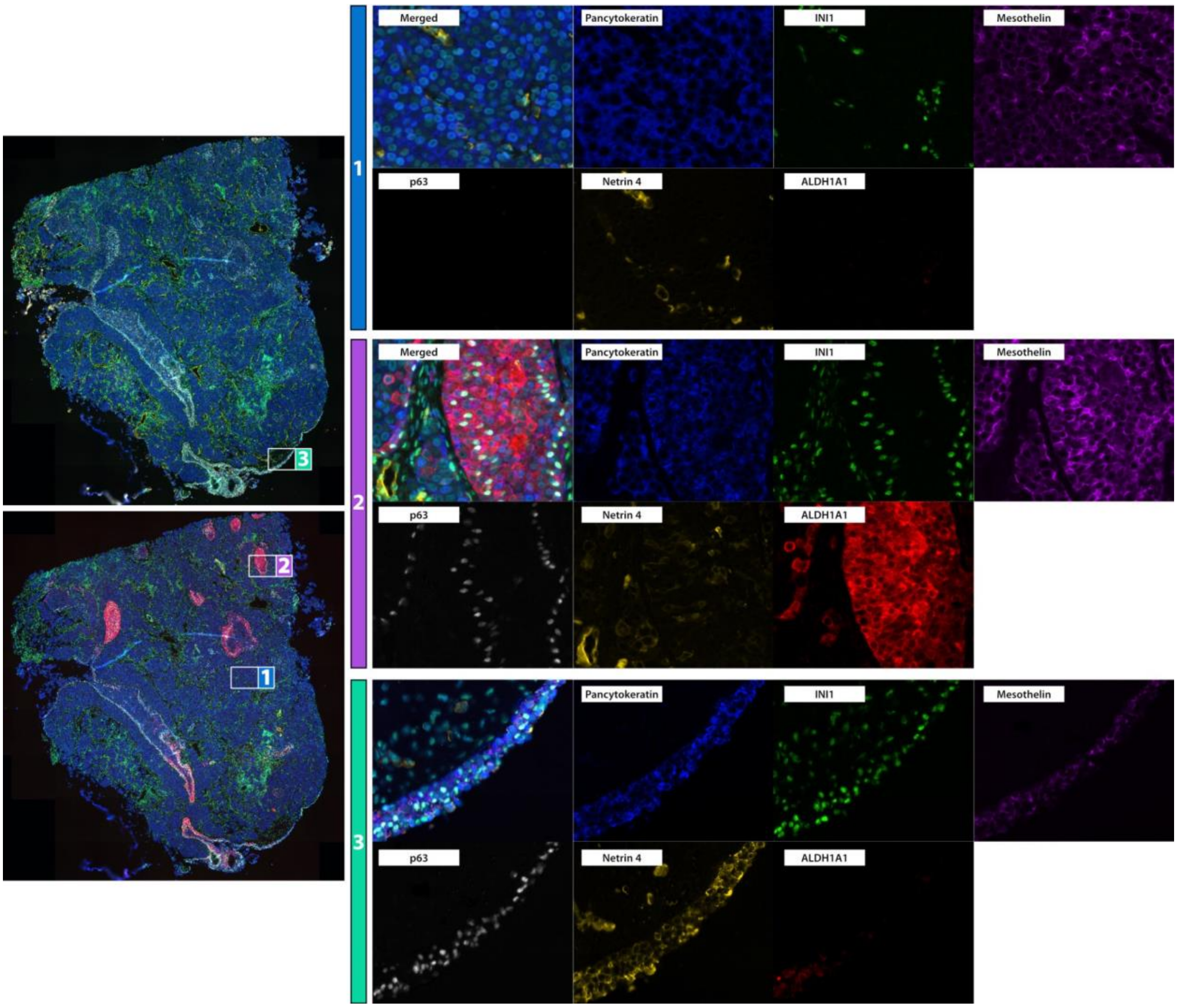

Updated Figure EV3 Now including mesothelin staining to assess staining pattern in different niches.

Comment 8: "Similarly, p63 and NTN4 staining is not shown in the final section. The authors state that none of the external samples displayed intermingling of p63-positive normal basal cells. It is unclear whether this means that p63 staining was absent or that the staining pattern differed from that observed in the index case. This point is important for understanding the potential presence of a normal basal epithelial population that may support tumor stem-like cells. As these stainings appear to have been performed, they should be shown in a supplementary figure."

Reply: We thank the reviewer for highlighting that the p63 and NTN4 immunostainings were not shown and for requesting clarification of our statement regarding the external cohort.

In the initial submission, we did not include a detailed description of p63 staining patterns across the external cohort, but we are happy to add this clarification now. As described before, in the additional SDSC cases, p63-positive non-neoplastic basal cells were not observed intermingling within the tumor. However, p63 expression was present in tumor cells in a subset of cases, with variable patterns (strong diffuse, patchy, or predominantly basal/peripheral). ALDH1A1 expression was independent of p63 status/pattern in the external cohort. We have added a new paragraph to the Results section detailing the

different expression patterns and included representative p63 seqIF images as a **new Figure EV5**.

The revised Results section now reads:

"In contrast to the index case, none of the additional SDSC cases showed intermingling of p63-positive non-neoplastic basal cells within the tumor. Instead, p63 immunoreactivity in tumor cells was observed in 7/14 cases (50%), a frequency in line with previous reports.1 We observed three distinct patterns: strong, diffuse nuclear staining in essentially all tumor cells (three cases; Figure EV5A), patchy staining in a subset of tumor cells (two cases; Figure EV5B), or predominantly basal/peripheral staining within tumor nests (two cases; Figure EV5C). The remaining 7/14 cases (50%) were p63-negative (Figure EV5D). Importantly, ALDH1A1 expression was not associated with p63 status or pattern and was observed in both p63-positive and p63-negative tumors."

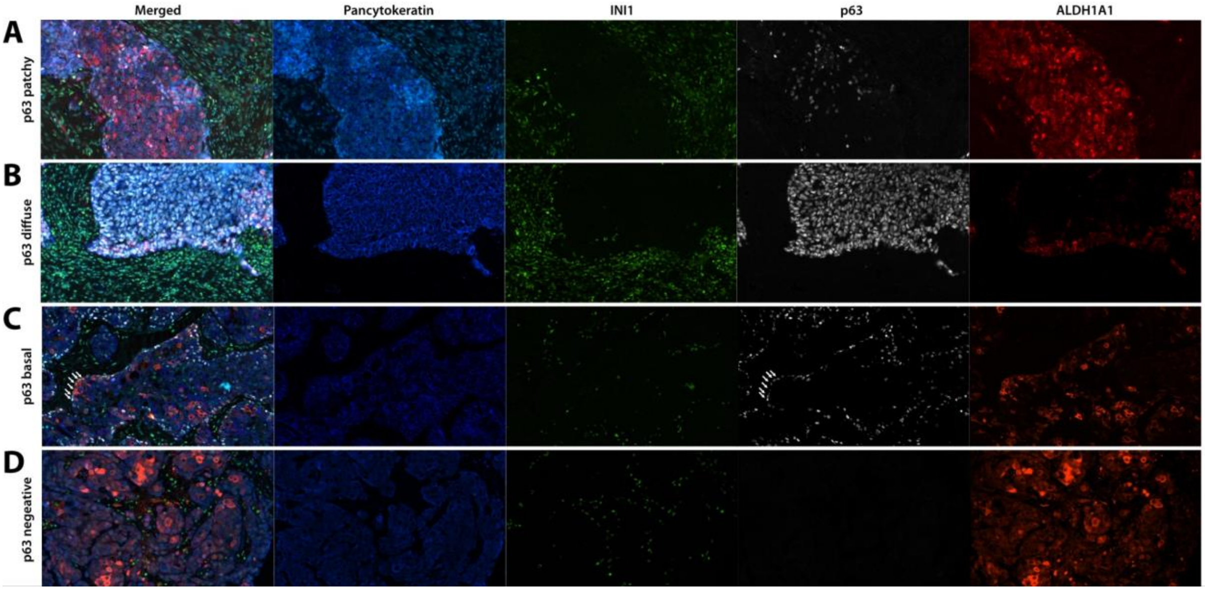

Figure EV5 Representative staining patterns of p63 in the retrospective cohort.
A Patchy expression of p63 in tumor cells (loss of nuclear INI1).
B Diffuse p63 expression in almost all tumor cells.
C Predominantly expression of p63 in tumor cells at the basal (near stroma) layer.
D No p63 expression in tumor cells.

Furthermore, we have added a **new Appendix Figure S10** with representative seqIF images of NTN4 expression in the retrospective cohort.

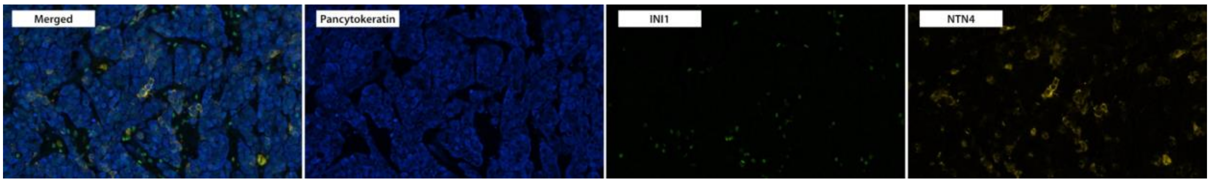

New Appendix Figure S10 Representative, patchy NTN4+ expression in tumor cells in the retrospective cohort without clear spatial organization.

Comment 9: “In the Discussion, the term "2D cell culture organoids" is used. The authors presumably mean "3D" organoid cultures.”

Reply: We thank the reviewer for making us aware of this typo which has been corrected in the revised version of the manuscript.

Comment 10: “The methods corresponding to Fig. 1A-B (H&E, immunocytochemistry and MRI) should be described.”

Reply: We thank the reviewer for this suggestion. We have expanded the Methods section to describe the procedures corresponding to Fig. 1A-B.

The updated sections now read:

“Representative clinical MRI images (Fig. 1A) were acquired on a Siemens MAGNETOM Aero 1.5T scanner (Siemens Healthineers, Germany) using a coronal T1-weighted spin-echo sequence. Acquisition parameters were: TR 526 ms, TE 8.8 ms, matrix 320 x 320, slice thickness 3 mm, slice spacing 3.3 mm.”

“Hematoxylin and eosin (H&E) staining was performed on an automated Tissue-Tek staining system (Sakura Finetek, Japan) according to the manufacturer’s instructions. Immunohistochemical analysis for INI1 was performed on an automated BenchMark XT staining platform (Ventana, USA) using the ready-to-use MRQ-27 antibody (Ventana, USA, RRID not available).”

Comment 11: “When relevant, software versions should be reported, and a link to the PathInfony pipeline should be provided. Appropriate references for the PROGENy and CollecTRI networks should also be included.”

Reply: We thank the reviewer for this helpful suggestion. We have now added the corresponding software versions throughout the Methods section and included appropriate references for PROGENy, CollecTRI, and decoupleR.

PathInfony consists of in-house Python wrapper scripts that are part of our routine molecular pathology bioinformatics pipeline. The framework is specifically tailored to integrate with our local IT infrastructure and pathology laboratory information systems and is therefore not readily transferable to other research environments. We have expanded the Methods section to describe all relevant analytical steps, databases, and filtering criteria used for variant annotation and prioritization and decided to omit explicit reference to PathInfony to ensure clarity and reproducibility for readers.

The revised Methods section now reads:

“Variants were annotated using VEP (Ensembl), ClinVar, and the BRCA Exchange database. Classification followed ACMG and VICC guidelines via the Franklin-Genoox database.”

(...)

"Analysis using the Illumina DRAGEN v4.2 RNA pipeline (BCL-to-FASTQ conversion, alignment, BAM/BAI generation) and ARRIBA enabled detection of fusion transcripts, which were annotated using MANE, HAVANA, UCSC, and COSMIC databases."

Comment 12: "The quality of the material for snRNA-seq is currently assessed using viable cell counts. This should be replaced with metrics appropriate for nuclei-based assays, such as nuclei integrity and nuclei counts."

Reply: We thank the reviewer for highlighting that our original quality assessment terminology was not appropriate for a nuclei-based assay. In the previous version, we mistakenly referred to a "viable cell count" which is of course not meaningful for FFPE-derived nuclei preparations. We have added the corresponding nuclei counts for all snRNAseq preparations as a new Table EV1. In addition, consistent with 10x Genomics recommendations for nuclei-based workflows, we performed visual/morphological quality assessment of the nuclei suspensions prior to downstream processing, confirming intact nuclear morphology with minimal debris and aggregation before proceeding.

The revised Methods section now reads:

"The number of intact nuclei was approximated with a Readycount Green/Red Cell Viability Stain, (ThermoFisher, USA) on the Countess 3 Cell Counter (ThermoFisher, USA; Table EV1). In addition, nuclei preparations were assessed by visual inspection of the counter images prior to downstream processing to confirm intact nuclear morphology and to exclude preparations with excessive debris or nuclear aggregation/clumping."

Comment 13: " In-text references to Figure 3 should be corrected."

Reply: We thank the reviewer for pointing this out. We identified a stray reference to Figure 3B-D in the context of the Sapanisertib-treated sample, which incorrectly implied that the referenced panels depicted treated rather than untreated cultured samples. This erroneous reference has been removed from the manuscript.

Comment 14: "Relevant abbreviations should be defined when first introduced, and gene name formatting (italicisation) should follow standard guidelines consistently throughout the manuscript."

Reply: We have revised the manuscript to ensure that all relevant abbreviations are defined at first mention (fixing UMAP, snRNAseq and RCTD). In addition, we standardized gene/protein nomenclature throughout the text and figure legends, applying italics consistently to human gene symbols and using non-italicized formatting for corresponding proteins in accordance with standard practice.

15th Apr 2026

Dear Prof. Jurmeister,

Thank you for the submission of your revised manuscript to EMBO Molecular Medicine. We have now heard back from the reviewer who was asked to re-evaluate your study. As you will see below, the reviewer is satisfied with the modifications made. Before we can formally accept your manuscript for publication, we would ask you to address the remaining issue mentioned by the reviewer.

On a more editorial level, please also do the following:

1. The following discrepancies in author names need to be resolved: Edgar Chimal (submission system) vs Edgar Chimal Calderon (manuscript), Benedikt Westphalen (submission system) vs Christoph Benedikt Westphalen (manuscript), Maximilian Leitheise (submission system) vs Maximilian Leitheiser (manuscript text), Lot Devise (submission system) vs Lot Devriese (manuscript).

2. Please merge the funding information with "Acknowledgments" and add project numbers to the "Acknowledgment" section and in our submission system, if available.

3. Please remove the "Author Contributions" section from the manuscript text.

4. Please move "the paper explained" to the main manuscript file and add section headings "problems", "results" and "impact". Please refer to our published articles for an example.

5. Table EV4: please include a separate sheet in the same file labeled 'Legend' containing the corresponding table legend.

6. Appendix: please add a Table of Contents on the title page with page numbers for all listed items.

7. Please add a missing panel callout for Fig EV1.

8. "Conflict of Interest" should be renamed to "Disclosure and competing interests statement".

9. The information in the "Ethics Approval and Consent to Participate" section should be incorporated into the relevant parts of the "Methods" section. The separate heading "Ethics Approval and Consent to Participate" should be removed.

10. Data availability: please make sure the datasets will be made publicly available upon the acceptance of the manuscript.

11. BIORENDER: remove from Acknowledgments and add as a dedicated section to the Methods, using this format:
Graphics:
(some of the... OR Figure #... OR synopsis) Graphics were created with BioRender.com.

12. Update the headings and order of the sections in the manuscript text to: Abstract / The Paper Explained / Introduction / Results /Discussion / Methods / Data Availability / Acknowledgements /Disclosure and Competing Interests Statement / References /Figure Legends / Expanded View Figure Legends

13. Please address the following issues in figure legends:
- Please note that the box plots need to be defined in terms of minima, maxima, centre, bounds of box and whiskers, and percentile in the legends of figures 4A
- Please note that information related to n is missing in the legend of figure 4A
- Please note that the scale bar is missing for figures EV5 A-D

I look forward to receiving a new revised version of your manuscript as soon as possible.

Kind regards,
Jingyi

Jingyi Hou
Senior Editor
EMBO Molecular Medicine

*** Instructions to submit your revised manuscript ***

*** PLEASE NOTE *** As part of the EMBO Publications transparent editorial process initiative (see our Editorial at https://www.embopress.org/doi/pdf/10.1002/emmm.201000094), EMBO Molecular Medicine will publish online a Review Process File to accompany accepted manuscripts.

In the event of acceptance, this file will be published in conjunction with your paper and will include the anonymous referee reports, your point-by-point response and all pertinent correspondence relating to the manuscript. If you do NOT want this file to be published, please inform the editorial office at contact@embomolmed.org.

*****

When preparing your revised manuscript, please refer to our guidelines: https://link.springer.com/journal/44321/submission-guidelines#cms-Revised-submissions. We perform an initial quality control of all revised manuscripts before re-review; failure to include requested items will delay the evaluation of your revision.

***** Reviewer's comments *****

Referee #2 (Comments on Novelty/Model System for Author):

This is a revised version of the manuscript in which the authors addressed all previous comments and considerably improved the analysis and clarity of the presented data.

Referee #2 (Remarks for Author):

I would like to commend the authors for the thorough approach in addressing the comments. I read the additional analysis and findings with great interest. Could the authors check figure EV5 and the associated text description for panels A and B (I believe they are reversed)?

Point-by-point response

Reviewer comments are displayed in blue.
Author replies are shown in black.
Citations from the revised main manuscript are written in italics.

Reviewer #2

Comment 1: " Could the authors check figure EV5 and the associated text description for panels A and B (I believe they are reversed)?"

Reply: We sincerely thank the reviewer for the very positive feedback. We have double checked Figure EV5 and indeed found that panels A and B were reversed. We have modified the figure accordingly. No changes to the manuscript text have been necessary.

16th Apr 2026

Dear Prof. Jurmeister,

We are pleased to inform you that your manuscript is accepted for publication and is now being sent to our publisher to be included in the next available issue of EMBO Molecular Medicine.

Your manuscript will be processed for publication by EMBO Press. It will be copy edited and you will receive page proofs prior to publication. Please note that you will be contacted by Springer Nature Author Services to complete licensing and payment information.

You may qualify for financial assistance for your publication charges - either via a Springer Nature fully open access agreement or an EMBO initiative. Check your eligibility: https://link.springer.com/journal/44321/how-to-publish-with-us

Should you be planning a Press Release on your article, please get in contact with embo_production@springernature.com as early as possible in order to coordinate publication and release dates.

If you have any questions, please do not hesitate to contact the Editorial Office. Thank you for your contribution to EMBO Molecular Medicine.

Sincerely,
Jingyi

Jingyi Hou
Senior Editor
EMBO Molecular Medicine

>>> Please note that it is EMBO Molecular Medicine policy for the transcript of the editorial process (containing referee reports and your response letter) to be published as an online supplement to each paper. If you do NOT want this, you will need to inform the Editorial Office via email immediately. More information is available here: https://link.springer.com/partners/embo-press/editorial-policies#Peer%20review

EMBO Press Author Checklist

Corresponding Author Name: Philipp Jurmeister	
Journal Submitted to: EMBO Molecular Medicine	
Manuscript Number: EMM-2026-23251	

USEFUL LINKS FOR COMPLETING THIS FORM
The EMBO Journal - Author Guidelines
EMBO Reports - Author Guidelines
Molecular Systems Biology - Author Guidelines
EMBO Molecular Medicine - Author Guidelines

Reporting Checklist for Life Science Articles (updated January
This checklist is adapted from Materials Design Analysis Reporting (MDAR) Checklist for Authors. MDAR establishes a minimum set of requirements in transparent reporting in the life sciences (see Statement of Task: 10.31222/osf.io/9sm4x). Please follow the journal's guidelines in preparing your
Please note that a copy of this checklist will be published alongside your article.

Abridged guidelines for figures
1. Data
The data shown in figures should satisfy the following conditions:
- the data were obtained and processed according to the field's best practice and are presented to reflect the results of the experiments in an accurate and unbiased manner.
- ideally, figure panels should include only measurements that are directly comparable to each other and obtained with the same assay.
- plots include clearly labeled error bars for independent experiments and sample sizes. Unless justified, error bars should not be shown for technical
- if n<5, the individual data points from each experiment should be plotted. Any statistical test employed should be justified.
- Source Data should be included to report the data underlying figures according to the guidelines set out in the authorship guidelines on Data

2. Captions
Each figure caption should contain the following information, for each panel where they are relevant:
- a specification of the experimental system investigated (eg cell line, species name).
- the assay(s) and method(s) used to carry out the reported observations and measurements.
- an explicit mention of the biological and chemical entity(ies) that are being measured.
- an explicit mention of the biological and chemical entity(ies) that are altered/varied/perturbed in a controlled manner.
- the exact sample size (n) for each experimental group/condition, given as a number, not a range;
- a description of the sample collection allowing the reader to understand whether the samples represent technical or biological replicates (including how many animals, litters, cultures, etc.).
- a statement of how many times the experiment shown was independently replicated in the laboratory.
- definitions of statistical methods and measures:
  - common tests, such as t-test (please specify whether paired vs. unpaired), simple χ2 tests, Wilcoxon and Mann-Whitney tests, can be unambiguously identified by name only, but more complex techniques should be described in the methods section;
  - are tests one-sided or two-sided?
  - are there adjustments for multiple comparisons?
  - exact statistical test results, e.g., P values = x but not P values < x;
  - definition of 'center values' as median or average;
  - definition of error bars as s.d. or s.e.m.

Please complete ALL of the questions below.
Select "Not Applicable" only when the requested information is not relevant for your study.

Materials

Newly Created Materials	Information included in the manuscript?	In which section is the information available? (Reagents and Tools Table, Materials and Methods, Figures, Data Availability Section)
New materials and reagents need to be available; do any restrictions apply?	Not Applicable	

Antibodies	Information included in the manuscript?	In which section is the information available? (Reagents and Tools Table, Materials and Methods, Figures, Data Availability Section)
For **antibodies** provide the following information:<br>- Commercial antibodies: RRID (if possible) or supplier name, catalogue number and or/clone number<br>- Non-commercial: RRID or citation	Yes	Materials and Methods

DNA and RNA sequences	Information included in the manuscript?	In which section is the information available? (Reagents and Tools Table, Materials and Methods, Figures, Data Availability Section)
**Short novel DNA or RNA including primers, probes**: provide the sequences.	Not Applicable	

Cell materials	Information included in the manuscript?	In which section is the information available? (Reagents and Tools Table, Materials and Methods, Figures, Data Availability Section)
**Cell lines:** Provide species information, strain. Provide accession number in repository **OR** supplier name, catalog number, clone number, and/**OR** RRID.	Not Applicable	
**Primary cultures:** Provide species, strain, sex of origin, genetic modification status.	Yes	Material and Methods
Report if the cell lines were recently **authenticated** (e.g., by STR profiling) and tested for mycoplasma contamination.	Not Applicable	

Experimental animals	Information included in the manuscript?	In which section is the information available? (Reagents and Tools Table, Materials and Methods, Figures, Data Availability Section)
**Laboratory animals or Model organisms:** Provide species, strain, sex, age, genetic modification status. Provide accession number in repository **OR** supplier name, catalog number, clone number, **OR** RRID.	Not Applicable	
**Animal observed in or captured from the field:** Provide species, sex, and age where possible.	Not Applicable	
Please detail **housing and husbandry conditions**.	Not Applicable	

Plants and microbes	Information included in the manuscript?	In which section is the information available? (Reagents and Tools Table, Materials and Methods, Figures, Data Availability Section)
**Plants:** provide species and strain, ecotype and cultivar where relevant, unique accession number if available, and source (including location for collected wild specimens).	Not Applicable	
**Microbes:** provide species and strain, unique accession number if available, and source.	Not Applicable	

Human research participants	Information included in the manuscript?	In which section is the information available? (Reagents and Tools Table, Materials and Methods, Figures, Data Availability Section)
If collected and within the bounds of privacy constraints report on age, sex and gender or ethnicity for all study participants.	Yes	Material and Methods, Results

Core facilities	Information included in the manuscript?	In which section is the information available? (Reagents and Tools Table, Materials and Methods, Figures, Data Availability Section)
If your work benefited from core facilities, was their service mentioned in the acknowledgments section?	Not Applicable	

Design

Study protocol	Information included in the manuscript?	In which section is the information available? (Reagents and Tools Table, Materials and Methods, Figures, Data Availability Section)
If study protocol has been **pre-registered, provide DOI in the manuscript**. For clinical trials, provide the trial registration number **OR** cite DOI.	Not Applicable	
Report the **clinical trial registration number** (at ClinicalTrials.gov or equivalent), where applicable.	Not Applicable	

Laboratory protocol	Information included in the manuscript?	In which section is the information available? (Reagents and Tools Table, Materials and Methods, Figures, Data Availability Section)
Provide DOI OR other citation details if **external detailed step-by-step protocols** are available.	Not Applicable	

Experimental study design and statistics	Information included in the manuscript?	In which section is the information available? (Reagents and Tools Table, Materials and Methods, Figures, Data Availability Section)
Include a statement about **sample size** estimate even if no statistical methods were used.	Yes	Material and Methods
Were any steps taken to minimize the effects of subjective bias when allocating animals/samples to treatment (e.g. **randomization procedure**)? If yes, have they been described?	Not Applicable	
Include a statement about **blinding** even if no blinding was done.	Yes	Material and Methods
Describe **inclusion/exclusion criteria** if samples or animals were excluded from the analysis. Were the criteria pre-established?<br><br>If sample or data points were omitted from analysis, report if this was due to attrition or intentional exclusion and provide justification.	Not Applicable	
For every figure, are **statistical tests** justified as appropriate? Do the data meet the assumptions of the tests (e.g., normal distribution)? Describe any methods used to assess it. Is there an estimate of variation within each group of data? Is the variance similar between the groups that are being statistically compared?	Not Applicable	

Sample definition and in-laboratory replication	Information included in the manuscript?	In which section is the information available? (Reagents and Tools Table, Materials and Methods, Figures, Data Availability Section)
In the figure legends: state number of times the experiment was **replicated** in laboratory.	Yes	Material and Methods
In the figure legends: define whether data describe **technical or biological replicates**.	Yes	Material and Methods

Ethics

Ethics	Information included in the manuscript?	In which section is the information available? (Reagents and Tools Table, Materials and Methods, Figures, Data Availability Section)
Studies involving **human participants**: State details of **authority granting ethics approval** (IRB or equivalent committee(s), provide reference number for approval.	Yes	Ethics Approval and Consent to Participate
Studies involving **human participants**: Include a statement confirming that **informed consent** was obtained from all subjects and that the experiments conformed to the principles set out in the WMA Declaration of Helsinki and the Department of Health and Human Services Belmont Report.	Yes	Ethics Approval and Consent to Participate
Studies involving **human participants: For** publication of **patient photos**, include a statement confirming that consent to publish was obtained.	Not Applicable	
Studies involving experimental **animals**: State details of **authority granting ethics approval** (IRB or equivalent committee(s), provide reference number for approval. Include a statement of compliance with ethical regulations.	Not Applicable	
Studies involving **specimen and field samples:** State if relevant **permits** obtained, provide details of authority approving study; if none were required, explain why.	Not Applicable	

Dual Use Research of Concern (DURC)	Information included in the manuscript?	In which section is the information available? (Reagents and Tools Table, Materials and Methods, Figures, Data Availability Section)
Could your study fall under dual use research restrictions? Please check biosecurity documents and list of **select agents and toxins** (CDC): https://www.selectagents.gov/sat/list.htm	Not Applicable	
If you used a select agent, is the security level of the lab appropriate and reported in the manuscript?	Not Applicable	
If a study is subject to dual use research of concern regulations, is the name of the **authority granting approval and reference number** for the regulatory approval provided in the manuscript?	Not Applicable	

Reporting

The MDAR framework recommends adoption of discipline-specific guidelines, established and endorsed through community initiatives. Journals have their own policy about requiring specific guidelines and recommendations to complement MDAR.

Adherence to community standards	Information included in the manuscript?	In which section is the information available? (Reagents and Tools Table, Materials and Methods, Figures, Data Availability Section)
State if relevant guidelines or checklists (e.g., **ICMJE, MIBBI, ARRIVE, PRISMA**) have been followed or provided.	Not Applicable	
For **tumor marker prognostic studies,** we recommend that you follow the **REMARK** reporting guidelines (see link list at top right). See author guidelines, under 'Reporting Guidelines'. Please confirm you have followed these guidelines.	Not Applicable	
For **phase II and III randomized controlled trials**, please refer to the **CONSORT** flow diagram (see link list at top right) and submit the CONSORT checklist (see link list at top right) with your submission. See author guidelines, under 'Reporting Guidelines'. Please confirm you have submitted this list.	Not Applicable	

Data Availability

Data availability	Information included in the manuscript?	In which section is the information available? (Reagents and Tools Table, Materials and Methods, Figures, Data Availability Section)
Have **primary datasets** been deposited according to the journal's guidelines (see 'Data Deposition' section) and the respective accession numbers provided in the Data Availability Section?	Yes	Data Availibility Section
Were **human clinical and genomic datasets** deposited in a public access-controlled repository in accordance to ethical obligations to the patients and to the applicable consent agreement?	Yes	Data Availibility Section, Ethics Approval and Consent to Participate
Are **computational models** that are central and integral to a study available without restrictions in a machine-readable form? Were the relevant accession numbers or links provided?	Not Applicable	
If publicly available data were reused, provide the respective **data citations in the reference list**.	Not Applicable	
